# Construction of a chromosome-scale long-read reference genome assembly for potato

**DOI:** 10.1093/gigascience/giaa100

**Published:** 2020-09-23

**Authors:** Gina M Pham, John P Hamilton, Joshua C Wood, Joseph T Burke, Hainan Zhao, Brieanne Vaillancourt, Shujun Ou, Jiming Jiang, C Robin Buell

**Affiliations:** Department of Plant Biology, Michigan State University, 612 Wilson Road, East Lansing, MI 48824, USA; Department of Plant Biology, Michigan State University, 612 Wilson Road, East Lansing, MI 48824, USA; Department of Plant Biology, Michigan State University, 612 Wilson Road, East Lansing, MI 48824, USA; Department of Plant Biology, Michigan State University, 612 Wilson Road, East Lansing, MI 48824, USA; Department of Plant Biology, Michigan State University, 612 Wilson Road, East Lansing, MI 48824, USA; Department of Plant Biology, Michigan State University, 612 Wilson Road, East Lansing, MI 48824, USA; Department of Ecology, Evolution, and Organismal Biology, Iowa State University, 2200 Osborne Dr, Ames, IA 50011, USA; Department of Plant Biology, Michigan State University, 612 Wilson Road, East Lansing, MI 48824, USA; Department of Horticulture, Michigan State University, 1066 Bogue St, East Lansing, MI 48824, USA; MSU AgBioResearch, Michigan State University, 446 W. Circle Drive, East Lansing, MI 48824, USA; Department of Plant Biology, Michigan State University, 612 Wilson Road, East Lansing, MI 48824, USA; MSU AgBioResearch, Michigan State University, 446 W. Circle Drive, East Lansing, MI 48824, USA; Plant Resilience Institute, Michigan State University, 612 Wilson Road, East Lansing, MI 48824, USA

**Keywords:** long-read, chromosome-scale, reference genome, potato

## Abstract

**Background:**

Worldwide, the cultivated potato, *Solanum tuberosum* L., is the No. 1 vegetable crop and a critical food security crop. The genome sequence of DM1–3 516 R44, a doubled monoploid clone of *S. tuberosum* Group Phureja, was published in 2011 using a whole-genome shotgun sequencing approach with short-read sequence data. Current advanced sequencing technologies now permit generation of near-complete, high-quality chromosome-scale genome assemblies at minimal cost.

**Findings:**

Here, we present an updated version of the DM1–3 516 R44 genome sequence (v6.1) using Oxford Nanopore Technologies long reads coupled with proximity-by-ligation scaffolding (Hi-C), yielding a chromosome-scale assembly. The new (v6.1) assembly represents 741.6 Mb of sequence (87.8%) of the estimated 844 Mb genome, of which 741.5 Mb is non-gapped with 731.2 Mb anchored to the 12 chromosomes. Use of Oxford Nanopore Technologies full-length complementary DNA sequencing enabled annotation of 32,917 high-confidence protein-coding genes encoding 44,851 gene models that had a significantly improved representation of conserved orthologs compared with the previous annotation. The new assembly has improved contiguity with a 595-fold increase in N50 contig size, 99% reduction in the number of contigs, a 44-fold increase in N50 scaffold size, and an LTR Assembly Index score of 13.56, placing it in the category of reference genome quality. The improved assembly also permitted annotation of the centromeres via alignment to sequencing reads derived from CENH3 nucleosomes.

**Conclusions:**

Access to advanced sequencing technologies and improved software permitted generation of a high-quality, long-read, chromosome-scale assembly and improved annotation dataset for the reference genotype of potato that will facilitate research aimed at improving agronomic traits and understanding genome evolution.

## Data Description

### Background

**Figure 1:**
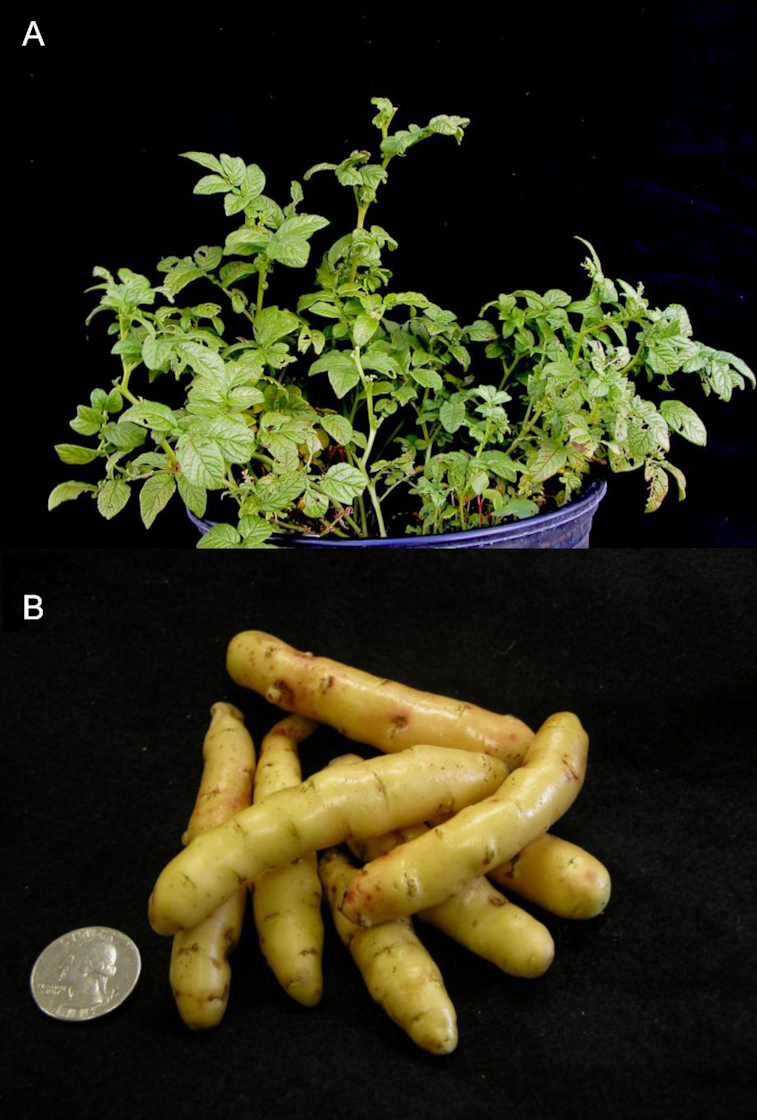
Doubled monoploid potato clone, DM1–3 516 R44. (a) Aboveground tissues and (b) tubers from the doubled monoploid potato clone, DM1–3 516 R44. Photos courtesy of Joseph Coombs.

The genome of the vegetable crop potato (*Solanum tuberosum* L., NCBI:txid4113) was published in 2011 by the Potato Genome Sequencing Consortium (PGSC) using a whole-genome shotgun sequencing approach [1]. At that time, Illumina sequencing was a newly available approach with high accuracy and throughput relative to previously available technologies. The reference genome was generated from the doubled monoploid clone, DM1–3 516 R44 (hereafter referred to as DM; Fig.   1), to reduce assembly difficulties due to the heterozygous and polyploid nature of tetraploid potato. The PGSC DM genome was assembled using a combination of 36 nucleotide (nt) reads from the Illumina Genome Analyzer platform and scaffolded using longer end sequence reads from fosmid and bacterial artificial chromosome clones generated using Sanger sequencing technology. This resulted in a highly fragmented genome assembly, with 90% of the assembly contained in 443 super-scaffolds with an N90 super-scaffold length of 359 kb and an N50 contig length of 31.4 kb [1]. With access to additional genetic maps and comparative data with tomato, the ordering, orientation, and anchoring of the initial PGSC assembly to the 12 chromosomes of potato was improved, yielding v4.03 of the DM genome [2]. DM v4.03 was then supplemented by the addition of new, unscaffolded contigs (v4.04) [3] (Table 1) generated through whole-genome sequencing and assembly of unaligned reads.

**Table 1:** Assembly metrics of the DM 1–3 R44 v4 and v6 assemblies

Parameter	v4.03^a^	v4.04^b^	v6.1^c^
Total assembly size, Mb	773.0	884.1	741.6
Total non-gapped size, Mb	676.3	728.7	741.5
Contig N50 size, bp	31,914	29,071	17,312,182
Total contig No.	60,068	170,833	1,382
Scaffold N50 size, bp	1,344,915	1,344,915	59,670,755
Scaffold No.	14,853	14,853	288

a PGSC contigs and scaffolds downloaded from NCBI: AEWC01000001-AEWC01060068; JH137791-JH152643 [1, 2].

b DM v4.04 is composed of v4.03 plus an additional 110,765 unanchored contigs (55.7 Mb) [3].

c The DM v6.1 scaffolds are composed of the 12 chromosome-scale pseudomolecules and 276 unanchored scaffolds.

The published DM sequence has undoubtedly served as a valuable resource in the plant genomics and potato genetics community as indicated by numerous publications that used the sequence (e.g., [3–13]). However, its quality and potential is limited by the technology that was available at the time of its publication; new technologies and approaches for genome sequencing and assembly, including linked reads, long-read sequencing, and chromatin contact map–based strategies [14] present new opportunities to improve upon the sequence of the potato genome. In this Data Note, the doubled monoploid clone DM was sequenced using long-read sequencing on the Oxford Nanopore Technologies (ONT) platform and assembled into highly contiguous pseudochromosomes using Hi-C scaffolding data. The final assembly, DM v6.1, improves upon contiguity in comparison with DM v.4.04, with longer contigs, fewer gaps, and more contiguous sequence, allowing for improved accuracy in future studies on potato genome biology, especially those requiring accurate intergenic sequence.

### DNA isolation, library construction, and sequencing

DM plants were grown in Murashige and Skoog (MS) medium (bioWORLD, Dublin, OH, Cat No. 3,063,014), shoots harvested, and flash frozen in liquid nitrogen. Nuclei were isolated following the Workman et al. [15] protocol with a genome size–dependent spin speed of 2,950*g*; a total of 6.2 g of shoot tissue was split across 6 separate nuclei isolations. Modifications to the protocol include squeezing the homogenate through 5 layers of Miracloth instead of gravity filtering alone and 2 washes with nuclear isolation buffer. DNA was isolated from nuclei using the Nanobind Plant Nuclei Big DNA—Alpha Version kit (Circulomics, Baltimore, MD, Cat No. NB-900–801-01) following the Nanobind Plant Nuclei Big DNA Kit Handbook v0.17 (May 2018). DNA libraries were prepared using the ONT SQK-LSK109 Ligation Sequencing kit (Oxford, UK). Six libraries were prepared and sequenced on 6 separate R9 ONT flow cells (1 FLO-MIN106 flow cell, 5 FLO-MIN106 Rev D flow cells). DNA repair and end preparation (New England BioLabs, Ipswich, MA, Cat Nos. E7546 and M6630) were performed with an input of 1 μg of DNA. The repair and end preparation reaction were incubated for 5–45 minutes at 20°C and 5–45 minutes at 60°C. The reaction was cleaned using Agencourt AMPure XP beads (Beckman Coulter, Brea, CA, Cat No. A63880) with an incubation time of 5–10 minutes on a rotator mixer and eluted for 2–5 minutes. Ligation of adapters to the prepared DNA was performed at room temperature for 10–60 minutes. The ligation reaction was cleaned using Agencourt AMPure XP beads on a rotator mixer with an incubation time of 5–10 minutes with an elution time of 10 minutes. Sequencing was performed on an ONT MinION (Oxford, UK, Cat No. MIN-101B) (MinION, RRID:SCR_017985) with the current release of MinKNOW (version 1.15.0). Sequencing was run for 48–92 hours (Supplementary Table S1). DNA was also isolated from young leaf following a modified CTAB protocol (2% cetyl trimethylammonium bromide [CTAB]), 100 mM Tris, 1.4 M sodium chloride, 20 mM ethylenediaminetetraacetic acid (EDTA), 1% 2-mercaptoethanol) [16]. An Illumina TruSeq DNA Nano whole-genome shotgun library was constructed for use in error correction and sequenced on an Illumina HiSeq 2500 (Illumina HiSeq 2500 System, RRID:SCR_016383) in paired-end mode, generating 150 nt reads (Supplementary Table S1). Hi-C library construction, DNA extraction, and library preparation were completed by Phase Genomics as described previously [17] and sequenced at the University of Minnesota Genomics Center (Supplementary Table S1).

### Generation of a long-read, chromosome-scale assembly for DM

The sequenced nanopore whole-genome shotgun sequencing libraries were base-called using Guppy (v3.2.2+9fe0a78  [18]) on an Amazon Web Services p3.2xlarge NVIDIA Tesla V100 GPU instance with the following parameters: –flowcell FLO-MIN106 –kit SQK-LSK109 -q 0 –qscore_filtering –trim_strategy dna –calib_detect. The reads that passed the base caller quality filter were then filtered with seqtk v1.3 (seqtk, RRID:SCR_018927) [19] to remove reads <10 kb (seq -A -L 10 000), yielding a final set of 1,050,302 reads with a total size of 38.2 Gb and ∼45× coverage (Supplementary Table S2). Contigs were assembled from the final set of nanopore reads using Flye v2.5 (Flye, RRID:SCR_017016) [20] with the parameters –nano-raw -g 850m -i 0. The initial assembly was then polished with the final set of nanopore reads using 4 iterations of Racon v1.3.2 (Racon, RRID:SCR_017642) [21]. For each iteration, the reads were mapped to the assembly using minimap2 v2.17 (minimap2, RRID:SCR_018550) [22] with the parameter -x map-ont, then polished with the read alignments using Racon with the -u parameter set. The assembly was further polished using the final set of long reads using 2 rounds of Nanopolish v0.11.1 (Nanopolish, RRID:SCR_016157) [23]. Reads were mapped with minimap2 v2.17 [22] with the parameters -ax map-ont and the alignments converted to BAM with Samtools v1.9 (Samtools, RRID:SCR_002105) [24]. An updated consensus VCF file was generated using nanopolish variants –consensus -x 5000 and the polished assembly generated using the VCF file with nanopolish vcf2fasta. Final polishing was performed with an Illumina whole-genome shotgun sequencing library (PEP_AA_01) using 3 rounds of Pilon v1.23 (Pilon, RRID:SCR_014731) [25]. The Illumina reads were processed by Cutadapt v2.5 (Cutadapt, RRID:SCR_011841) [26] to remove adapters and to trim low-quality regions with the parameters -n 2 -m 100 -q 10. For each iteration, the cleaned reads were aligned to the assembly using BWA-MEM v0.7.17 (BWA-MEM, RRID:SCR_010910) [27], duplicate alignments marked with Picard MarkDuplicates v2.3.4 (MarkDuplicates, RRID:SCR_006525) [28], and the alignments sorted with Picard SortSam v2.3.4 (SortSam, RRID:SCR_006525), all using default parameters. Pilon was run using the “–fix bases” option. The polished contigs are composed of 1,382 contigs with a total size of 745.6 Mb with an N50 contig size of 17.3 Mb and a maximum contig length of 42.1 Mb (Table 1).

To construct chromosome-scale pseudomolecules, the Hi-C library was first processed using the juicer.sh pipeline from the Juicer package (git commit 6403a27) (Juicer, RRID:SCR_017226) [29]. The pseudomolecules were then assembled with the run-asm-pipeline.sh from the 3D-DNA pipeline (git commit 529ccf4) (3D-DNA, RRID:SCR_017227) [30] and the results visualized in Juicebox v1.11.08 [31] (Supplementary Fig. S1). To detect contamination, the pseudomolecules and unanchored scaffolds were split into 10-kb windows and searched against the NCBI nt [32] database using Centrifuge v1.0.4-beta (Centrifuge, RRID:SCR_016665) [33] with the parameters –min-hitlen 200 -f -x nt. Examination of the report generated by Centrifuge-kreport indicated that there were no regions identified as non-Viridiplantae contaminants. To identify contigs from organellar genomes, pseudomolecules and unanchored scaffolds were searched against the DM chloroplast genome (JF772172.1), the draft DM mitochondrion genome (JF772170.2), and a complete *S. tuberosum* mitochondrion genome (MN114537.1, MN114538.1, MN114539.1) using blastn v2.9.0 (blastn, RRID:SCR_001598) [34]. Fifteen unanchored scaffolds were identified as originating from the organellar genomes and were removed from the assembly. In total, 731,287,687 bp were placed on the 12 chromosomes, leaving 10,297,348 bp unanchored. Overall, the new v6.1 assembly improves upon the previous DM assembly in terms of contiguity, with a 595-fold increase in N50 contig size, 99% reduction in number of contigs, and a 44-fold increase in N50 scaffold size (Tables 1 and 2).

**Table 2:** Chromosome lengths and gap (N) content in DM v4.04 and v6.1

Chromosome	DM v4.04					DM v6.1				
	Total chromosome length (bp)	Total sequence length (bp)	% Sequence	Total gap length (bp)	% Gaps	Total chromosome length (bp)	Total sequence length (bp)	% Sequence	Total gap length (bp)	% Gaps
chr01	88,663,952	77,894,594	87.85	10,769,358	12.15	88,591,686	88,579,186	99.99	12,500	0.01
chr02	48,614,681	42,696,816	87.83	5917,865	12.17	46,102,915	46,100,415	99.99	2,500	0.01
chr03	62,290,286	53,928,846	86.58	8361,440	13.42	60,707,570	60,704,570	100	3,000	0.00
chr04	72,208,621	62,203,573	86.14	10,005,048	13.86	69,236,331	69,230,831	99.99	5,500	0.01
chr05	52,070,158	46,610,373	89.51	5459,785	10.49	55,599,697	55,591,197	99.98	8,500	0.02
chr06	59,532,096	51,644,783	86.75	7887,313	13.25	59,091,578	59,085,578	99.99	6,000	0.01
chr07	56,760,843	49,550,308	87.30	7210,535	12.70	57,639,317	57,635,317	99.99	4,000	0.01
chr08	56,938,457	49,300,183	86.59	7638,274	13.41	59,226,000	59,217,000	99.98	9,000	0.02
chr09	61,540,751	53,891,571	87.57	7649,180	12.43	67,600,300	67,594,300	99.99	6,000	0.01
chr10	59,756,223	52,349,496	87.61	7406,727	12.39	61,044,151	61,037,651	99.99	6,500	0.01
chr11	45,475,667	40,128,174	88.24	5347,493	11.76	46,777,387	46,772,387	99.99	5,000	0.01
chr12	61,165,649	53,902,062	88.12	7263,587	11.88	59,670,755	59,658,755	99.98	12,000	0.02
Total pseudomolecules	725,017,384	634,100,779	87.46	90,916,605	12.54	731,287,687	731,207,187	99.99	80,500	0.01
Unanchored sequences	159,090,912	94,595,563	59.46	64,495,349	40.54	10,297,348	10,289,348	99.92	8,000	0.08
Total assembly	884,108,296	728,696,342	82.42	155,411,954	17.58	741,585,035	741,496,535	99.99	88,500	0.01

### Assessment of the contiguity and accuracy of the v6.1 assembly

To assess completeness and accuracy of the v6.1 assembly, ∼458 million paired-end reads from a whole-genome Illumina sequencing library (PEP_AA_01; Supplementary Table S1) were mapped to the v6.1 and v4.04 genome assembly. Cutadapt v2.8 (Cutadapt, RRID:SCR_011841) [26] was used to remove adapters and trim low-quality bases (Q < 20) prior to alignment to the genome assemblies using BWA-MEM v0.7.16a (BWA-MEM, RRID:SCR_010910) [27]. Alignment rates to v6.1 were excellent, with 98.05% of the whole-genome shotgun reads aligned and properly paired, relative to 96.70% in DM v4.04 (Supplementary Table S3), with 6.84% of the whole-genome shotgun reads aligned to v6.1 with a MAPQ score of equal to 0 versus 10.13% in v4.04. BUSCO v4.0.5 (BUSCO, RRID:SCR_015008) [35] software was used to estimate representation of genic space in the DM v6.1 genome assembly [35]. Of 1,614 total BUSCO orthologs in the embryophyta_odb10 database, 1,579 complete BUSCO orthologs (97.9% completeness; 1,544 single copy and 35 duplicated) were detected, with 18 fragmented and 17 missing BUSCO orthologs (Supplementary Table S4). These results are nearly identical to that of DM v4.04, demonstrating that the DM v.4.04 assembly provided robust representation of the genic space, even though it was generated using short-read technologies and was highly fragmented. The heterozygosity of the genome was estimated by counting canonical *k*-mers (*k* = 21) from the cleaned Illumina whole-genome shotgun library (PEP_AA_01) using Jellyfish2 v2.2.10 (Jellyfish, RRID:SCR_005491) [36]. The *k*-mer count histogram was analyzed by the online version of GenomeScope (GenomeScope, RRID:SCR_017014) [37] and the heterozygosity of the genome was estimated at 0.0383% (Supplementary Fig. S2).

**Figure 2:**
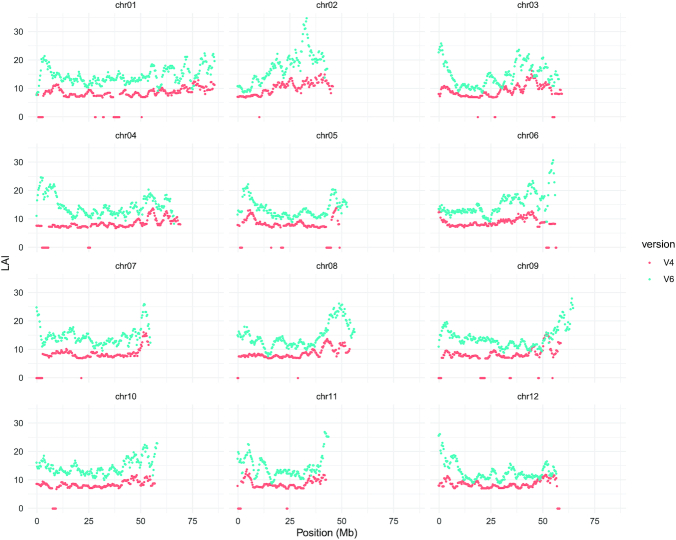
Genome-wide LTR Assembly Index (LAI) [38] scores for DM assembly v.4.04 (V4) and v.6.1 (V6). LAI was calculated for 3-Mb sliding windows with a 300-kb step size.

The Long Terminal Repeat (LTR) Assembly Index (LAI) [38] metric was used to evaluate assembly continuity in DM v6.1 and v4.04. Intact LTR retrotransposons of the 2 assemblies were identified using LTRharvest v1.6.1 (LTRharvest, RRID:SCR_018970) [39], LTR_FINDER_parallel v1.1 (LTR_FINDER_parallel, RRID:SCR_018969) [40], and LTR_retriever v2.8.7 (LTR_retriever, RRID:SCR_017623) [41]. LTR sequence libraries of DM v6.1 and v4.04 were combined using the cleanup_nested.pl script from the LTR_retriever package with parameters -cov 0.95 -minlen 80 -miniden 80 -t 36. The LAI program was executed using parameters -q -t 36 -totLTR 51.76 -iden 91.59 -unlock to generate an overall LAI score for assemblies of DM v6.1 and v4.04. Higher LAI scores correspond to more complete genome assemblies because a greater number of intact LTR retrotransposons are identified in these cases. The DM v4.04 genome had an LAI score of 7.87, a score that characterizes it as a draft genome assembly. In comparison, DM v6.1 has an improved LAI score of 13.56, placing it in the category of reference genome quality. Genomes of reference quality have an LAI score between 10 and 20; other examples of reference quality genomes include *Arabidopsis thaliana* TAIR10 (LAI = 14.9), *Fragaria vesca* v4.1 (LAI = 16.9), and *Solanum pennellii* (LAI = 14.8) [38]. The LAI score was also calculated for 3-Mb sliding windows with a 300-kb step size, showing noticeably higher scores in DM v6.1 relative to v4.04 (Fig. 2).

**Figure 3:**
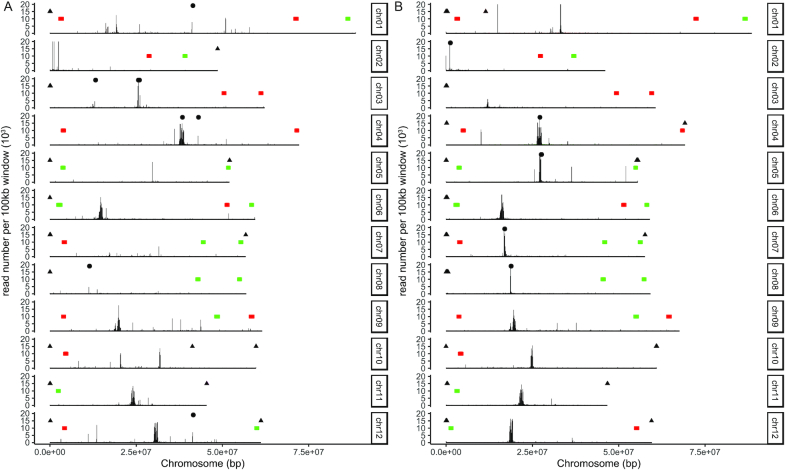
Distribution of subtelomeric repeat sequences, centromeric repeat sequences, CENH3 ChIP-seq alignments, and oligonucleotide fluorescent *in situ* hybridization (oligo-FISH) probes. (A) Distribution of features on DM v4.04 assembly. (B) Distribution of features on DM v6.1 assembly. Red and green rectangles represent the positions of the 2 “barcode” oligo-FISH probes [42]. For CENH3 ChIP-seq reads, chromosomes were divided into 100-kb windows and CENH3 read number in each window was calculated and plotted [45]. Circles represent centromeric repeats [45]. Triangles represent subtelomeric repeats [55].

Two “barcode” oligonucleotide fluorescent *in situ* hybridization (oligo-FISH) probes, which mark 26 regions on the 12 chromosomes, have been used to characterize potato karyotypic variation [42], as well as the evolution of chromosomes in distantly related *Solanum* species. We aligned the oligo-FISH probes to v6.1 using BWA-MEM v0.7.12-r1039 (BWA-MEM, RRID:SCR_010910) [27] to confirm the correct assembly of the 12 chromosomes. Each chromosome has a specific hybridization pattern (i.e., a barcode) and all 12 chromosomes of the v6.1 assembly had an alignment pattern consistent with cytogenetic evidence (Fig. 3).

**Figure 4:**
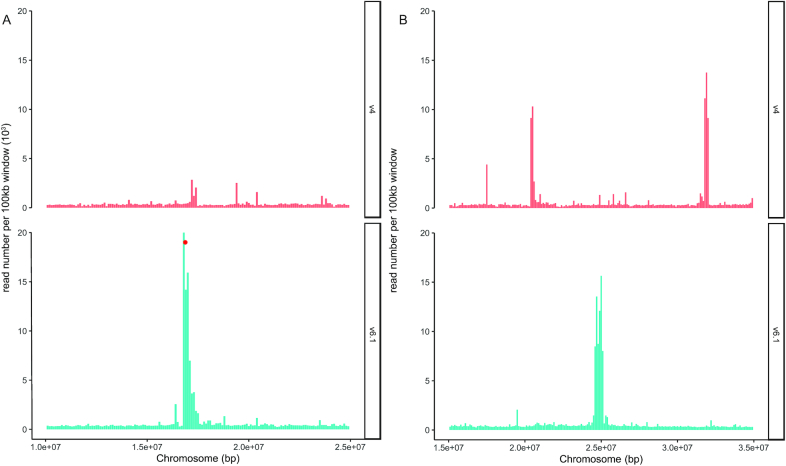
Improved assembly of the centromeric regions in DM v6.1. (A) CENH3 read distribution on centromere 7. (B) CENH3 read distribution on centromere 10. Chromosomes were divided into 100-kb windows and the CENH3 ChIP-seq read number [45] in each window was calculated and plotted. Red dots represent centromeric repeats. Upper panel shows the CENH3 ChIP-seq read distribution in the DM v4.04 assembly; lower panel shows the distribution in the DM v6.1 assembly.

A genetic map constructed from a DM × RH F1 population consisting of 190 individuals was used to validate the order and orientation of scaffolds placed within the DM v6.1 pseudomolecules [43]. The map was generated using 2,621 single-nucleotide polymorphism markers placed within 654 recombination bins and manually adjusted to eliminate incorrect bins. Vmatch v2.3.0 (Vmatch, RRID:SCR_018968) [44] with 200 nt of flanking sequence around each marker was used in alignments to DM v6.1 to check the concordance of the assembly with the genetic map; 2,444 (93.2%) of the markers perfectly aligned to v6.1, with an additional 24 markers aligning if 1 mismatch was permitted. Overall, the alignments demonstrate a high degree of congruence between the physical and genetic distances (Supplementary Fig. S3), with the exception of chromosome 12, which is inverted in the v6.1 assembly relative to the genetic map. The DM × RH genetic map, constructed in 2015, was ordered on the basis of marker position on v4.04. In v6.1, chromosome 12 has 5.76 Mb additional sequence compared with v4.04 because while chromosome 12 of v4.04 is 61.2 Mb in length, 7.26 Mb are Ns (Table 2). To further confirm that the short and long arms of chromosome 12 are correctly oriented in v6.1, we annotated the position of the centromeres using CENH3 ChIP-seq data obtained from a previous study [45]. ChIP-seq reads were aligned to the DM v6.1 assembly with BWA-MEM v0.7.12-r1039 (BWA-MEM, RRID:SCR_010910) [27] using default parameters. Chromosomes were divided into 100-kb windows, and read numbers in each window calculated using BEDTools v2.28.0 (BEDTools, RRID:SCR_006646) [46] to determine the distribution of sequences associated with CENH3 protein along the length of each chromosome. In comparison with v4.04, more centromeres are represented in v6.1 and *Cen12* is properly positioned on the short arm of v6.1 chromosome 12 (Fig. 3, Supplementary Table S5). The improved contiguity of v6.1 also enabled improved delineation of other centromeres, as shown for *Cen7*, which was absent in v4.04 while a clear CENH3 peak is detectable in v6.1 (Fig. 4A). In v4.04, *Cen10* was split into 2 regions, and in v6.1, it is assembled into a contiguous sequence (Fig. 4B). The size of potato centromere, which is defined by the size of the CENH3-binding domain, is ≥1,000 kb [45]. It worth noting that the CENH3-binding domains in some v6.1 centromeres were only several hundred kilobase pairs (Supplementary Table S5). These centromeres likely contain long stretches of repetitive sequences associated with CENH3 nucleosomes, and the small CENH3 binding domain in v6.1 is likely due to the collapse of repetitive sequences on these centromeres during assembly [47].

**Figure 5:**
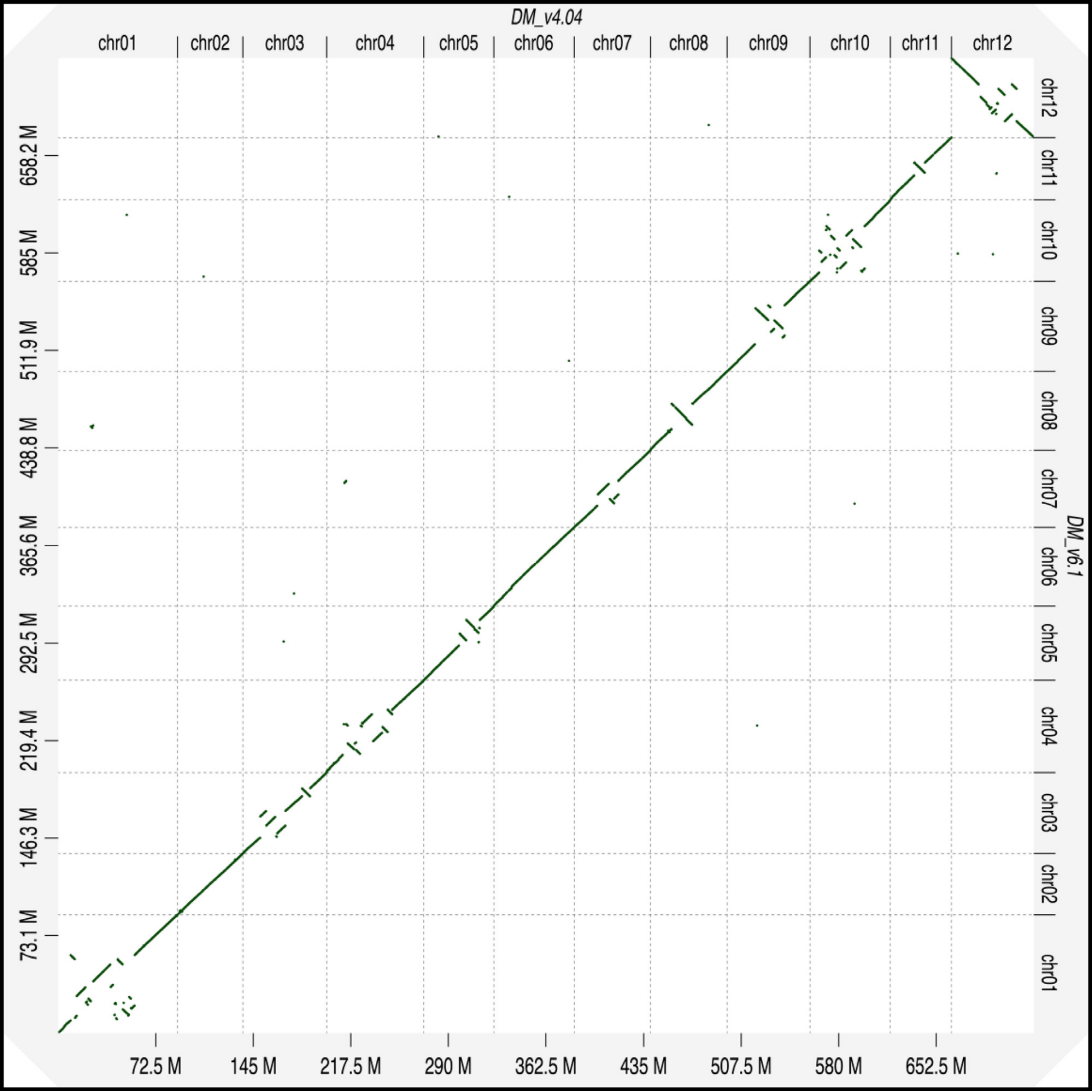
Whole-genome alignment of the DM v4.04 vs v6.1 DM genome assemblies. Whole-genome alignments of the long-read, chromosome-scale DM v6.1 assembly with the DM 4.04 genome assembly using D-GENIES reveals concordance in the euchromatic arms but misassemblies in the pericentromeric regions.

To better depict the improved contiguity and accuracy of v6.1 relative to v4.04, D-GENIES (D-GENIES, RRID:SCR_018967) [48] was used to generate whole-genome alignments between the 2 assemblies. As shown in Fig. 5, there are large blocks of collinearity between the 2 assemblies in the euchromatic arms. However, for every chromosome except chromosome 6 and chromosome 2, which is acrocentric and in which the short arm is almost entirely composed of the nucleolar organizing region, mis-assemblies were apparent in the pericentromeric regions. Because DM v4.04 was assembled into short contigs that were scaffolded using bacterial artificial chromosome and fosmid end sequences coupled with a low-density genetic map, it is not surprising that heterochromatic regions, which are not only repetitive but also low in genetic marker density, had assembly challenges. For DM v6.1, access to long reads coupled with chromatin-contact data highlights the power of advanced technologies to improve genome assembly accuracy. Overall, the reduced contig number, increased contig length, and improved accuracy of DM v6.1 exceeds the quality of DM v4.04.

### Repetitive landscape in DM

A custom repeat library (CRL) was generated using RepeatModeler2 v2.0.1 (RepeatModeler2, RRID:SCR_015027) [49] with the final contigs. Protein-coding genes were removed from the CRL using ProtExcluder v1.2 [50] by first searching the CRL against the alluniRefprexp070416 plant protein database [51] using blastx v2.4.0 (blastx, RRID:SCR_001653) [52] with an e-value cut-off of 1e−10 and processing the results using ProtExcluder.pl. The CRL was then combined with Viridiplantae repeats from RepBase v20150807 [53] to generate the final CRL. The genome assembly was repeat-masked using the final CRL and RepeatMasker v4.1.0 (RepeatMasker, RRID:SCR_012954) [54] using the parameters -e ncbi -s -nolow -no_is -gff (Supplementary Table S6). In total, 495.7 Mb (66.8.%) of the DM v6.1 assembly was repeat-masked with the final CRL. Relative to v4.04, substantially more of each repetitive sequence class was identified, which is attributable to the longer contiguous sequence that enabled more robust detection of repeats and consistent with the increased LAI metric.

Potato is unusual in that the centromeres of 5 chromosomes (*Cen4, Cen6, Cen10, Cen11*, and *Cen12*) lack typical centromere-specific satellite repeats and, instead, are composed of single- or low-copy sequences resembling neocentromeres [45]. This contrasts with 6 centromeres (*Cen1, Cen2, Cen3, Cen5, Cen7*, and *Cen8*) that contain megabase arrays of satellite repeats. Interestingly, the satellite repeats for these 6 centromeres are unique to individual chromosomes, some of which are derived from retrotransposons. Centromeric repeat sequences from Gong et al. [45] were aligned to v4.04 and v6.1 genomes with BLAST v2.3.28 (BLAST, RRID:SCR_004870) [52] with alignments with >99% identity over 95% of the query length retained. Expected centromere-specific repeats were identified in *Cen2, Cen5*, and *Cen7* in v.6.1 but not in v4.04 (Fig. 3). In addition, the centromere-specific repeats were detected only in a single region in each respective chromosome in v.6.1. These results show significantly improved assembly of the centromeric sequences of v6.1 compared with v4.04. Two subtelomeric repeats have also been characterized in potato [55]. These 2 repeats were aligned to v6.1 and hits with >90% identity over 80% of the query length were retained. We identified these repeats on 16 chromosomal ends in v.6.1 whereas 15 chromosomal ends were identified in v4.04 (Fig. 3).

### Annotation

To facilitate annotation of gene models, ONT complementary DNA (cDNA) sequences were generated from DM. DM was grown under a 16-hour day length in tissue culture and RNA was isolated from whole tissue-culture plants using a modified hot borate method [56]. DNA contaminants were removed using the Ambion Turbo DNase Kit (Thermofisher Scientific, Waltham, MA) and Dynabeads mRNA DIRECT Purification Kit (Thermofisher Scientific, Waltham, MA) was used to isolate messenger RNA (mRNA). An ONT PCR-cDNA Sequencing library was constructed using the SQK-PCS109 kit (ONT, Oxford, UK) with the following modifications: input was increased to 5 ng of mRNA, GC Melt Reagent (Takara Bio, Inc., Kusatsu, Shiga, Japan) was included at a final concentration of 0.5 M during reverse transcription and PCR, PrimeScript reverse transcriptase (Takara Bio, Inc., Kusatsu, Shiga, Japan) was used for reverse transcription, 14 PCR cycles were performed with an extension time of 5 minutes, all Hula mixer steps were performed by hand, and the adapter ligation period was extended to 15 minutes with gentle mixing every 5 minutes. The completed library was sequenced using the MinION (MIN-101B) platform with an R9 FLO-MIN106 Rev D flow cell in 2 runs to maximize the yield of reads, the first connected to an Apple Macintosh computer running MinKNOW v3.5.5 and the second connected to an ONT MinIT running MinKNOW v3.6.3 and MinIT 19.2.1. The sequenced ONT cDNA library was base-called using Guppy 3.6.0+98ff765  [18] on an Amazon Web Services p3.2xlarge NVIDIA Tesla V100 GPU instance with the parameters –flowcell FLO-MIN106 –kit SQK-PCS109 -q 0 –qscore_filtering –trim_strategy none –calib_detect. The reads that passed the base caller quality filter were then processed with Pychopper v.2.4.0 (Pychopper, RRID:SCR_018966) [57] to identify full-length cDNA reads. The full-length and rescued cDNA reads were filtered with seqtk (seq -L 500) (seqtk, RRID:SCR_018927) [19] to remove reads <500 nt. The filtered cDNA reads were aligned to the genome assembly with minimap2 v2.2.17 (minimap2, RRID:SCR_018550) with the parameters -a -x splice -uf -G 5000; 5,783,924 (99.98%) of the 5,784,833 filtered reads aligned to the DM assembly. The cDNA alignments were assembled using Stringtie2 v2.1.2 (Stringtie2, RRID:SCR_016323) [58] (-L -m 500) and the assembled transcript sequences extracted with gffread v0.11.7 (gffread, RRID:SCR_018965) [59]. Illumina TruSeq Stranded mRNA-Seq libraries previously prepared from DM leaf (NCBI SRA SRX2023785 and SRX2023786) and tuber (NCBI SRA SRX2023789 and SRX2023798) tissues were used to generate RNA-Seq transcript assemblies for gene model refinement. Reads were first cleaned using Cutadapt v2.9 (Cutadapt, RRID:SCR_011841) [26] with the parameters -n 2 -m 100 -q 10, aligned to the genome assembly using HISAT2 v2.2.0 (HISAT2, RRID:SCR_015530) [60] with the parameters –max-intronlen 5000 –rna-strandness RF –no-unal –dta, and assembled using Stringtie v2.1.1 (Stringtie, RRID:SCR_016323) [58] with the parameter –rf and the assembled transcript sequences extracted with gffread v0.11.7 (gffread, RRID:SCR_018965) [59]. Both the leaf and tuber RNA-seq datasets were obtained from asymptomatic plants infected with potato virus X, and overall, reduced alignment rates to the DM v6.1 genome were observed in the leaf (67.31%) and tuber (66.43%) RNA-seq libraries.

The BRAKER2 (git commit 6219573) (BRAKER2, RRID:SCR_018964) [61] gene prediction pipeline was used to train Augustus v3.3.3 (Augustus, RRID:SCR_008417) [62] using GeneMark-ET v4.57 (GeneMark-ET, RRID:SCR_011930) [63] and the RNA-Seq alignments to generate *ab initio* gene predictions. The BRAKER2 pipeline was run using the command line: braker.pl –species = DM_v6_1 –gff3 –softmasking –UTR = off –bam {RNA-seq.alns.bam}. *Ab initio* gene predictions were refined using PASA2 v2.4.1 (PASA2, RRID:SCR_014656) [64] with the RNA-Seq and ONT cDNA transcript assemblies as evidence. Two rounds of annotation comparison were performed resulting in a set of 52,953 working gene models representing 40,652 loci. To identify high-confidence gene models, the working gene model set was searched against the PFAM database v32 (PFAM, RRID:SCR_004726) [65] with the hmmscan tool in HMMER v3.2.1 (HMMER, RRID:SCR_005305) [66] with a cut-off of –domE 1e-3 -E 1e-5 to identify gene models encoding a Pfam domain. Gene expression abundances (transcripts per million [TPM]) were generated using the leaf and tuber mRNA-Seq reads using Kallisto v0.46.0 (Kallisto, RRID:SCR_016582) [67].

High-confidence gene models were defined as having a TPM value > 0 in ≥1 RNA-Seq library and/or having a PFAM domain match. Gene models that were partial or had matches to transposable element–related PFAM domains were excluded from the high-confidence model set. A total of 32,917 loci encoding 44,851 gene models are contained within the high-confidence set (Supplementary Table S7). To assign functional annotation to the gene models, searches using the predicted proteins were performed with the *Arabidopsis* proteome (TAIR10; RRID:SCR_004618) [68], the PFAM database v32 (PFAM, RRID:SCR_004726) [65], and the Swiss-Prot plant proteins (release 2015_08) (Universal Protein Resource, RRID:SCR_002380). Search results were processed in the same order, and the function of the first hit encountered was assigned to the gene model. The quality of the annotation was evaluated using BUSCO [35], and both the working and high-confidence gene sets in v6.1 provided excellent representation of the conserved orthologs, with 93.5% complete in the working set and 93.0% complete in the high-confidence set (Supplementary Table S4). In contrast, the v4.04 annotation provided 74.6% complete BUSCO orthologs.

## Conclusions

Using improved sequencing technologies, the genome sequence of the reference potato genotype DM was vastly improved in contiguity relative to the previous release, DM v4.04. Version 6.1 of the DM genome assembly represents 87.8% of the estimated genome, with 595-fold increase in N50 contig size, 99% reduction in number of contigs, and a 44-fold increase in N50 scaffold size. Importantly, 731.2 Mb of the 741.6-Mb assembly is non-gapped and anchored to the 12 chromosomes, indicating a high degree of contiguity that was reflected in a “reference quality” LAI score, demonstrating the ability of advanced sequencing methods to assemble large contiguous regions of a medium-sized plant genome. With access to full-length cDNA sequences, 32,917 high-confidence protein-coding genes encoding 44,851 gene models were annotated, which provided a substantial improvement in representation of conserved orthologs compared with the previous annotation that will facilitate future studies in potato biology, genetics, and genomics.

## Availability of Supporting Data and Materials

The clone, DM1–3516 R44, is available through the United States Department of Agriculture Potato Genebank via PI GS 233 [69]. The raw genomic sequences and ONT cDNA are available in the NCBI SRA database under BioProject PRJNA636376. The genome assembly, annotation, CRL, and BUSCO results are available in the *GigaScience* GigaDB [70], Dryad Digital Repository [71], and on Spud DB [72, 73] via a JBrowse installation and download page.

## Additional Files

Supplementary Figure S1. Hi-C contact map showing the inter- and intra-chromosomal chromatin interactions in DM v6.1. Inter-chromosomal chromatin interactions are off the diagonal axis and intra-chromosomal chromatin interactions are within the blue boxes. Each pixel represents the degree of interaction between each 1-Mb locus, with a dark red color indicating a greater number of reads involved in the interaction. The blue boxes represent the boundaries of each pseudomolecule, and individual scaffold boundaries are represented by the green boxes.

Supplementary Figure S2. Estimation of heterozygosity of the DM genome as determined by GenomeScope. The DM genome has an estimated heterozygosity rate of 0.0383% using a *k-*mer of 21.

Supplementary Figure S3. Mapping of the DM × RH F1 population markers to the (a) DM v4.04 and the (b) DM v6.1 assembly. Flanking sequence (200 nt) of the markers was used for sequence alignments to the assembly using Vmatch (Vmatch, RRID:SCR_018968) [74]. The y-axis shows the map location in centimorgans, and the x-axis shows the physical location in megabases.

Supplementary Table S1. Sequence datasets used in this study. Total reads for Oxford Nanopore Technologies sequencing are passed reads after base-calling.

Supplementary Table S2. Oxford Nanopore Technologies whole-genome shotgun sequence reads used in the DM v6.1 assembly.

Supplementary Table S3. Illumina whole-genome shotgun sequence read mapping statistics.

Supplementary Table S4. BUSCO [35] results of the DM genome assemblies and annotation.

Supplementary Table S5. Centromere positions in the DM v6.1 assembly.

Supplementary Table S6. Repetitive sequence content in v4.04 and v6.1 DM 1–3516 R44 genome assemblies.

Supplementary Table S7. DM v6.1 gene annotation summary.

giaa100_GIGA-D-20-00167_Original_Submission

giaa100_GIGA-D-20-00167_Revision_1

giaa100_Response_to_Reviewer_Comments_Original_Submission

giaa100_Reviewer_1_Report_Original_Submission Jian-Feng Mao, Ph.D. -- 7/7/2020 Reviewed

giaa100_Reviewer_2_Report_Original_Submission Alexis Sullivan -- 8/10/2020 Reviewed

giaa100_Supplemental_Files

## Abbreviations

BLAST: Basic Local Alignment Search Tool; bp: base pairs; BUSCO: Benchmarking Universal Single-Copy Orthologs; cDNA: complementary DNA; ChIP-Seq: chromatin immunoprecipation sequencing; CRL: custom repeat library; Gb: gigabase pairs; kb: kilobase pairs; LAI: LTR Assembly Index; LTR: long terminal repeat; Mb: megabase pairs; mRNA: messenger RNA; NCBI: National Center for Biotechnology Information; nt: nucleotide; oligo-FISH: oligonucleotide fluorescent *in situ* hybridization; ONT: Oxford Nanopore Technologies; PASA: Program to Assemble Spliced Alignments; PGSC: Potato Genome Sequencing Consortium; RNA-Seq: RNA-sequencing; SRA: Sequence Read Archive; TPM: transcripts per million.

## Competing Interests

The authors declare that they have no competing interests.

## Authors’ Contributions

C.R.B. conceived the study. G.M.P., J.P.H., B.V., and J.C.W. performed the experiments. J.T.B., J.P.H., J.J., G.M.P., S.O., B.V., J.C.W., and H.Z. analyzed data. C.R.B., J.P.H., J.J., G.M.P., B.V., J.C.W., and H.Z. wrote the manuscript. All authors approved the final manuscript.
