# Supplementary material for: Construction of a chromosome-scale long-read reference genome assembly for potato
Source: Gigascience. 2020 Sep 23;9(9):giaa100. doi: 10.1093/gigascience/giaa100 (PMC7509475; doi:10.1093/gigascience/giaa100)
Supplement: giaa100_GIGA-D-20-00167_Original_Submission [file giaa100_giga-d-20-00167_original_submission.pdf]

GigaScience

Construction of a chromosome-scale long-read reference genome assembly for potato

--Manuscript Draft--

**Manuscript Number:**	GIGA-D-20-00167
**Full Title:**	Construction of a chromosome-scale long-read reference genome assembly for potato
**Article Type:**	Data Note
**Funding Information:**	PepsiCo (US) — Prof. C Robin Buell<br>USDA (MICL02431) — Prof. C Robin Buell<br>USDA (2017-67011-26038) — Dr Gina Pham<br>Directorate for Biological Sciences (IOS-1444514) — Dr Jiming Jiang

Abstract:

Background : Worldwide, the cultivated potato, Solanum tuberosum L. , is the number one vegetable crop and a critical food security crop. The genome sequence of DM1-3 516 R44, a doubled monoploid clone of S. tuberosum Group Phureja, was published in 2011 using a whole-genome shotgun sequencing approach with short read sequence data. Current advanced sequencing technologies now permit generation of near-complete, high-quality chromosome-scale genome assemblies at a minimal cost. Findings : Here, we present an updated version of the DM1-3 516 R44 genome sequence (v6.1) using Oxford Nanopore Technologies long reads coupled with proximity-by-ligation scaffolding (Hi-C) yielding a chromosome-scale assembly. The new (v6.1) assembly represents 741.6 Mb of sequence (87.8 %) of the estimated 844 Mb genome, of which, 741.5 Mb is non-gapped with 731.2 Mb anchored to the 12 chromosomes. Use of Oxford Nanopore Technologies full-length cDNA sequencing enabled annotation of 32,917 high-confidence protein-coding genes encoding 44,851 gene models that had a significantly improved representation of conserved orthologs compared to the previous annotation. The new assembly has improved contiguity with a 595-fold increase in N50 contig size, 99% reduction in the number of contigs, a 44-fold increase in N50 scaffold size, and an LTR Assembly Index score of 13.56, placing it in the category of reference genome quality. The improved assembly also permitted annotation of the centromeres via alignment to sequencing reads derived from CENH3 nucleosomes. Conclusions : Access to advanced sequencing technologies and improved software permitted generation of a high-quality, long-read, chromosome-scale assembly and improved annotation dataset for the reference genotype of potato that will facilitate research aimed at improving agronomic traits and understanding genome evolution.

**Corresponding Author:**	C Robin Buell<br>Michigan State University<br>East Lansing, Michigan UNITED STATES
**Corresponding Author Secondary Information:**	
**Corresponding Author's Institution:**	Michigan State University
**Corresponding Author's Secondary Institution:**	
**First Author:**	Gina Pham
**First Author Secondary Information:**	
**Order of Authors:**	Gina Pham
	John P Hamilton
	Joshua Wood
	Joseph T Burke
	Hainan Zhao

Powered by Editorial Manager® and ProduXion Manager® from Aries Systems Corporation

	Brieanne Vaillancourt
	Shujun Ou
	Jiming Jiang
	C Robin Buell
**Order of Authors Secondary Information:**	
**Additional Information:**	
**Question**	**Response**
Are you submitting this manuscript to a special series or article collection?	No
**Experimental design and statistics**<br><br>Full details of the experimental design and statistical methods used should be given in the Methods section, as detailed in our Minimum Standards Reporting Checklist. Information essential to interpreting the data presented should be made available in the figure legends.<br><br>Have you included all the information requested in your manuscript?	Yes
**Resources**<br><br>A description of all resources used, including antibodies, cell lines, animals and software tools, with enough information to allow them to be uniquely identified, should be included in the Methods section. Authors are strongly encouraged to cite Research Resource Identifiers (RRIDs) for antibodies, model organisms and tools, where possible.<br><br>Have you included the information requested as detailed in our Minimum Standards Reporting Checklist?	Yes
**Availability of data and materials**<br><br>All datasets and code on which the conclusions of the paper rely must be either included in your submission or	Yes

Powered by Editorial Manager® and ProduXion Manager® from Aries Systems Corporation

deposited in publicly available repositories (where available and ethically appropriate), referencing such data using a unique identifier in the references and in the “Availability of Data and Materials” section of your manuscript.<br><br>Have you have met the above requirement as detailed in our Minimum Standards Reporting Checklist?	

Manuscript Click here to download Manuscript DM_v13.docx

DATA NOTE

Construction of a chromosome-scale long-read reference genome assembly for potato

Gina M. Pham[1#], John P. Hamilton[1#], Joshua C. Wood[1], Joseph T. Burke[1], Hainan Zhao[1], Brieanne Vaillancourt[1], Shujun Ou[2], Jiming Jiang[1,3,5], and C. Robin Buell[1,4,5*]

[1]Department of Plant Biology, Michigan State University, East Lansing MI 48824 USA

[2]Department of Ecology, Evolution, and Organismal Biology, Iowa State University, Ames IA 50011 USA

[3]Department of Horticulture, Michigan State University, East Lansing MI 48824 USA

[4]Plant Resilience Institute, Michigan State University, East Lansing MI 48824 USA

[5]MSU AgBioResearch, Michigan State University, East Lansing MI 48824 USA

[#]These authors contributed equally to this work.

∗Correspondence address. C. Robin Buell, Department of Plant Biology, Michigan State University, 612 Wilson Road, East Lansing, MI 48824, USA, E-mail: buell@msu.edu

http://orcid.org/0000-0002-6727-4677

Keywords: long-read, chromosome-scale, reference genome, potato

Abstract

Background: Worldwide, the cultivated potato, *Solanum tuberosum* L., is the number one vegetable crop and a critical food security crop. The genome sequence of DM1-3 516 R44, a doubled monoploid clone of S. *tuberosum* Group Phureja, was published in 2011 using a whole-genome shotgun sequencing approach with short read sequence data. Current advanced sequencing technologies now permit generation of near-complete, high-quality chromosome-scale genome assemblies at a minimal cost. *Findings*: Here, we present an updated version of the DM1-3 516 R44 genome sequence (v6.1) using Oxford Nanopore Technologies long reads coupled with proximity-by-ligation scaffolding (Hi-C) yielding a chromosome-scale assembly. The new (v6.1) assembly represents 741.6 Mb of sequence (87.8 %) of the estimated 844 Mb genome, of which, 741.5 Mb is non-gapped with 731.2 Mb anchored to the 12 chromosomes. Use of Oxford Nanopore Technologies full-length cDNA sequencing enabled annotation of 32,917 high-confidence protein-coding genes encoding 44,851 gene models that had a significantly improved representation of conserved orthologs compared to the previous annotation. The new assembly has improved contiguity with a 595-fold increase in N50 contig size, 99% reduction in the number of contigs, a 44-fold increase in N50 scaffold size, and an LTR Assembly Index score of 13.56, placing it in the category of reference genome quality. The improved assembly also permitted annotation of the centromeres via alignment to sequencing reads derived from CENH3 nucleosomes. *Conclusions*: Access to advanced sequencing technologies and improved software permitted generation of a high-quality, long-read, chromosome-scale assembly and improved annotation dataset for the reference genotype of potato that will facilitate research aimed at improving agronomic traits and understanding genome evolution.

Data Description

Background

The genome of the vegetable crop potato (*Solanum tuberosum* L.) was published in 2011 by the Potato Genome Sequencing Consortium (PGSC) using a whole-genome shotgun sequencing approach [1]. At that time, Illumina sequencing was a newly available approach with high accuracy and throughput relative to previously available technologies. The reference genome was generated from the doubled monoploid clone, DM1-3 516 R44 (hereafter referred to as DM; Figure 1), to reduce assembly difficulties due to the heterozygous and polyploid nature of tetraploid potato. The PGSC DM genome was assembled using a combination of 36 nucleotide (nt) reads from the Illumina Genome Analyzer platform and scaffolded using longer end sequence reads from fosmid and bacterial artificial chromosome clones generated using Sanger sequencing technology. This resulted in a fragmented genome assembly, with 90% of the assembly contained in 443 super-scaffolds with an N90 super-scaffold length of 359 kb and an N50 contig length of 31.4 kb [1]. With access to additional genetic maps and comparative data with tomato, the ordering, orientation and anchoring of the initial PGSC assembly to the 12 chromosomes of potato was improved, yielding v4.03 of the DM genome [2]. DM v4.03 was then supplemented by the addition of new, unscaffolded contigs (v4.04) [3] (Table 1) generated through whole-genome sequencing and assembly of unaligned reads.

The published DM sequence has undoubtedly served as a valuable resource in the plant genomics and potato genetics community as indicated by numerous publications that utilized the sequence (e.g., [3-13]). However, its quality and potential is limited by the technology that was available at the time of its publication; new technologies and approaches for genome sequencing and assembly, including linked reads, long-read sequencing, and chromatin contact map-based strategies [14] present new opportunities to improve upon the sequence of the potato genome. In this Data Note, the doubled monoploid clone DM was sequenced using long-read sequencing on the Oxford Nanopore Technologies (ONT) platform and assembled into highly contiguous pseudochromosomes

using Hi-C scaffolding data. The final assembly, DM v6.1, improves upon contiguity in comparison to DM v.4.04, with longer contigs, fewer gaps, and more contiguous sequence, allowing for improved accuracy in future studies on potato genome biology, especially those requiring accurate intergenic sequence.

DNA isolation, library construction, and sequencing

DM plants were grown in Murashige and Skoog (MS) medium (bioWORLD, Dublin, OH, Cat # 3063014), shoots harvested, and flash frozen in liquid nitrogen. Nuclei were isolated following the Workman et al. [15] protocol with a genome size dependent spin speed of 2,950 x g; a total of 6.2 grams of shoot tissue was split across six separate nuclei isolations. Modifications to the protocol include squeezing the homogenate through five layers of Miracloth instead of gravity filtering alone and two washes with nuclear isolation buffer. DNA was isolated from nuclei using the Nanobind Plant Nuclei Big DNA – Alpha Version kit (Circulomics, Baltimore, MD, Cat # NB-900-801-01) following the Nanobind Plant Nuclei Big DNA Kit Handbook v0.17 (05/18). DNA libraries were prepared using the ONT SQK-LSK109 Ligation Sequencing kit (Oxford, UK). Six libraries were prepared and sequenced on six separate ONT flow cells (one FLO-MIN106 flow cell, five FLO-MIN106 Rev D flow cells). DNA repair and end-prep (New England BioLabs, Ipswich, MA, Cat #E7546 and Cat #M6630) were performed with an input of 1 μg of DNA. The repair and end-prep reaction were incubated for 5-45 minutes at 20 °C and 5-45 minutes at 60 °C. The reaction was cleaned using Agencourt AMPure XP beads (Beckman Coulter, Brea, CA, Cat #A63880) with an incubation time of 5-10 minutes on a rotator mixer and eluted for 2-5 minutes. Ligation of adapters to the prepared DNA was performed at room temperature for 10-60 minutes. The ligation reaction was cleaned using Agencourt AMPure XP beads on a rotator mixer with an incubation time of 5-10 minutes with an elution time of 10 minutes. Sequencing was performed on an ONT MinION (Oxford, UK, Cat # MIN-101B) with the current release of MinKNOW (version 1.15.0). Sequencing was run for 48-92 hours (Table S1). DNA was isolated from young leaf following a modified CTAB protocol (2% Cetyl trimethylammonium bromide; CTAB), 100 mM Tris, 1.4M Sodium chloride, 20 mM Ethylenediaminetetraacetic acid (EDTA), 1% 2-mercaptoethanol) [16].

An Illumina TruSeq DNA Nano whole-genome shotgun library was constructed for use in error correction and sequenced on an Illumina HiSeq 2500 in paired-end mode generating 150 nt reads (Table S1). Hi-C library construction, DNA extraction, and library preparation were completed by Phase Genomics as described previously [17] and sequenced at the University of Minnesota Genomics Center (Table S1).

Generation of a long-read, chromosome-scale assembly for DM

The sequenced nanopore whole-genome shotgun sequencing libraries were base-called using Guppy (v3.2.2+9fe0a78; [18]) on an Amazon Web Services p3.2xlarge NVIDIA Tesla V100 GPU instance with the parameters: --flowcell FLO-MIN106 --kit SQK-LSK109 -q 0 --qscore_filtering --trim_strategy dna --calib_detect. The reads that passed the base caller quality filter were then filtered with seqtk (v1.3, [19]) to remove reads less than 10kb (seq -A -L 10000) yielding a final set of 1,050,302 reads with a total size of 38.2 Gb (Table S2). Contigs were assembled from the final set of nanopore reads using Flye (v2.5, [20]) with the parameters --nano-raw -g 850m -i 0. The initial assembly was then polished with the final set of nanopore reads using four iterations of Racon (v1.3.2; [21]). For each iteration, the reads were mapped to the assembly using minimap2 (v2.17; [22]) with the parameter -x map-ont, then polished with the read alignments using Racon with the -u parameter set. The assembly was further polished using the final set of long reads using two rounds of Nanopolish (v0.11.1;[23]). Reads were mapped with minimap2 (v2.17; [22]) with the parameters (-ax map-ont) and the alignments converted to BAM with Samtools (v1.9; [24]). An updated consensus VCF file was generated using Nanopolish variants --consensus -x 5000 and the polished assembly generated using the VCF file with Nanopolish vcf2fasta. Final polishing was performed with an Illumina whole-genome shotgun sequencing library using three rounds of Pilon (v1.23; [25]). The Illumina reads were processed by Cutadapt (v2.5; [26]) to remove adapters and to trim low quality regions with the parameters: -n 2 -m 100 -q 10. For each iteration, the cleaned reads were aligned to the assembly using BWA-MEM (v0.7.17; [27]), duplicate alignments marked with Picard MarkDuplicates (v2.3.4; [28]), and the alignments sorted with Picard SortSam

(v2.3.4), all using default parameters. Pilon was run using the "--fix bases" option. The polished contigs are composed of 1,382 contigs with a total size of 745.6 Mb with an N50 contig size of 17.3 Mb and a maximum contig length of 42.1 Mb (Table 1).

To construct chromosome-scale pseudomolecules, the Hi-C library was first processed using the juicer.sh pipeline from the Juicer package (git commit 6403a27; [29]). The pseudomolecules were then assembled with the run-asm-pipeline.sh from the 3D-DNA pipeline (git commit 529ccf4; [30]) and the results visualized in Juicebox (v1.11.08; [31]; Figure S1). To detect contamination, the pseudomolecules and unanchored scaffolds were split into 10 kb windows and searched against the National Center for Biotechnology Information (NCBI) nt [32] database using Centrifuge (v1.0.4-beta; [33]) with the parameters (--min-hitlen 200 -f -x nt). Examination of the report generated by Centrifuge-kreport indicated there were no regions that were identified as non-Viridiplantae contaminants. To identify contigs from organellar genomes, pseudomolecules and unanchored scaffolds were searched against the DM chloroplast genome (JF772172.1), the draft DM mitochondrion genome (JF772170.2) and a complete *Solanum tuberosum* mitochondrion genome (MN114537.1, MN114538.1, MN114539.1) using blastn (v2.9.0; [34]). Fifteen unanchored scaffolds were identified as originating from the organellar genomes and were removed from the assembly. In total, 731,287,687 bp were placed on the 12 chromosomes leaving 10,297,348 bp unanchored. Overall, the new v6.1 assembly improves upon the previous DM assembly in terms of contiguity with a 595-fold increase in N50 contig size, 99% reduction in number of contigs, and a 44-fold increase in N50 scaffold size (Tables 1 and 2).

Assessment of the contiguity and accuracy of the v6.1 assembly

To assess completeness and accuracy of the v6.1 assembly, ~459 million paired-end reads from whole-genome Illumina sequencing were mapped to the v6.1 and v4.04 genome assembly. Cutadapt (v2.8; [26]) was used to remove adapters and trim low quality bases (Q < 20) prior to alignment to the genome assemblies using BWA-MEM (v0.7.16a; [27]). Alignment rates to v6.1 were excellent with 98.05% of the whole-genome

shotgun reads aligned and properly paired relative to 96.70% in DM v4.04 (Table S3) with 6.84% of the whole-genome shotgun reads aligned to v6.1 with a MAPQ score of equal to 0 versus 10.13% in v4.04. Benchmarking Universal Single-Copy Orthologs (BUSCO; [35]) v4.0.5 software was used to estimate representation of genic space in the DM v6.1 genome assembly [35]. Of 1,614 total BUSCO orthologs in the embryophyta_odb10 database, 1,579 complete BUSCO orthologs (97.9% completeness; 1,544 single copy and 35 duplicated) were detected with 18 fragmented and 17 missing BUSCO orthologs (Table S4). These results are nearly identical to that of DM v4.04, demonstrating that the DM v.4.04 assembly provided robust representation of the genic space, even though it was generated using short-read technologies and was highly fragmented.

The Long Terminal Repeat (LTR) Assembly Index (LAI) [36] metric was used to evaluate assembly continuity in DM v6.1 and v4.04. Intact LTR retrotransposons of the two assemblies were identified using LTRharvest (v1.6.1; [37]), LTR_FINDER_parallel (v1.1; [38]), and LTR_retriever (v2.8.7; [39]). LTR sequence libraries of DM v6.1 and v4.04 were combined using the cleanup_nested.pl script from the LTR_retriever package with parameters: -cov 0.95 -minlen 80 -miniden 80 -t 36. The LAI program was executed using parameters -q -t 36 -totLTR 51.76 -iden 91.59 -unlock to generate an overall LAI score for assemblies of DM v6.1 and v4.04. Higher LAI scores correspond to more complete genome assemblies, as a greater number of intact LTR retrotransposons are identified in these cases. The DM v4.04 genome had an LAI score of 7.87, a score that characterizes it as a draft genome assembly. In comparison, DM v6.1 has an improved LAI score of 13.56, placing it in the category of reference genome quality. Genomes of reference quality have an LAI score between 10 and 20; other examples of reference quality genomes include *Arabidopsis thaliana* TAIR10 (LAI = 14.9), *Fragaria vesca* v4.1 (LAI = 16.9), and *Solanum pennellii* (LAI = 14.8) [36]. The LAI score was also calculated in sliding 300 kb sliding windows, showing noticeably higher scores in DM v6.1 relative to v4.04 (Figure 2).

Two “barcode” oligonucleotide fluorescent *in situ* hybridization (Oligo-FISH) probes, which mark 26 regions on the 12 chromosomes, have been used to characterize potato karyotypic variation [40] as well as the evolution of chromosomes in *Solanum* species. We aligned the Oligo-FISH probes to v6.1 using BWA-MEM (v0.7.12-r1039; [27]) to confirm the correct assembly of the 12 chromosomes. Each chromosome has a specific hybridization pattern (i.e., a barcode) and all 12 chromosomes of the v6.1 assembly had an alignment pattern consistent with cytogenetic evidence (Figure 3).

A genetic map constructed from a DM x RH F1 population consisting of 190 individuals was used to validate the order and orientation of scaffolds placed within the DM v6.1 pseudomolecules [41]. The map was generated using 2,621 single nucleotide polymorphism markers placed within 654 recombination bins and manually adjusted to eliminate incorrect bins. Vmatch (v2.3.0; [42]) with 200 nt of flanking sequence around each marker was used in alignments to DM v6.1 to check concordance of the assembly with the genetic map; 2,444 (93.2%) of the markers perfectly aligned to v6.1 with an additional 24 markers aligning if one mismatch was permitted. Overall, the alignments demonstrate a high degree of congruence between the physical and genetic distances (Figure S2) with the exception of chromosome 12, which is inverted in the v6.1 assembly relative to the genetic map. The DM x RH genetic map, constructed in 2015, was ordered based on marker position on v4.04. In v6.1, chromosome 12 has 5.76 Mb additional sequence compared to v4.04 as while chromosome 12 of v4.04 is 61.2 Mb in length, 7.26 Mb are Ns (Table 2). To further confirm that the short and long arm of chromosome 12 are correctly oriented in v6.1, we annotated the position of the centromeres using CENH3 chromatin immunoprecipation-seq (ChIP-seq) data obtained from a previous study [43]. ChIP-seq reads were aligned to the DM v6.1 assembly with BWA-MEM (v0.7.12-r1039; [27]) using default parameters. Chromosomes were divided into 100 kb windows, and read numbers in each window calculated using BEDTools (v2.28.0; [44])to determine the distribution of CENH3 protein association along the length of each chromosome. In comparison to v4.04, more centromeres are

represented in v6.1 and *Cen12* is properly positioned on the short arm of v6.1 chromosome 12 (Figure 3, Table S5). The improved contiguity of v6.1 also enabled improved delineation of other centromeres as shown for *Cen7*, which was absent in v4.04 while a clear CENH3 peak is detectable in v6.1 (Figure 4a). In v4.04, *Cen10* was split into two regions and in v6.1, it is assembled into a contiguous sequence (Figure 4b). The size of potato centromere, which is defined by the size of the CENH3-binding domain, is at least 1,000 kb [43]. It worth noting that the CENH3-binding domains in some v6.1 centromeres were only several hundred kilobase pairs (Table S5). These centromeres likely contain long stretches of repetitive sequences associated with CENH3 nucleosomes and the small CENH3 binding domain in v6.1 is likely due to the collapse of repetitive sequences on these centromeres during assembly [45].

To better depict the improved contiguity and accuracy of v6.1 relative to v4.04, D-GENIES [46] was used to generate whole-genome alignments between the two assemblies. As shown in Figure 5, there are large blocks of collinearity between the two assemblies in the euchromatic arms. However, for every chromosome except chromosome 6 and chromosome 2, which is acrocentric and in which the short arm is almost entirely composed of the nucleolar organizing region, mis-assemblies were apparent in the pericentromeric regions. As DM v4.04 was assembled into short contigs that were scaffolded using bacterial artificial chromosome and fosmid end sequences coupled with a low-density genetic map, it is not surprising that heterochromatic regions, which are not only repetitive but also low in genetic marker density, had assembly challenges. For DM v6.1, access to long reads coupled with chromatin-contact data highlight the power of advanced technologies to improve genome assembly accuracy. Overall, the reduced contig number, increased contig length, and improved accuracy of DM v6.1 exceeds the quality of DM v4.04.

Repetitive landscape in DM

A custom repeat library (CRL) was generated using RepeatModeler2 (v2.0.1; [47]) with the final contigs. Protein-coding genes were removed from the CRL using ProtExcluder

(v1.2; [48]) by first searching the CRL against the alluniRefprexp070416 plant protein database [49] using blastx (v2.4.0; [50]) with an a e-value cutoff of 1e-10 and processing the results using ProtExcluder.pl. The CRL was then combined with Viridiplantae repeats from RepBase (v20150807;[51]) to generate the final CRL. The genome assembly was repeat-masked using the final CRL and RepeatMasker (v4.1.0;[52]) using the parameters: -e ncbi -s -nolow -no_is -gff (Table S6). In total, 495.7 Mb (66.8.%) of the DM v6.1 assembly was repeat-masked with the final CRL. Relative to v4.04, substantially more of each repetitive sequence class was identified attributable to the longer contiguous sequence that enabled more robust detection of repeats and consistent with the increased LAI metric.

Potato is unusual in that the centromeres of five chromosomes (*Cen4, Cen6, Cen10, Cen11,* and *Cen12*) lack typical centromere-specific satellite repeats and instead, are composed of single- or low- copy sequences resembling neocentromeres [43]. This contrasts with six centromeres (*Cen1, Cen2, Cen3, Cen5, Cen7* and *Cen8*) that contain megabase arrays of satellite repeats. Interestingly, the satellite repeats for these six centromeres are unique to individual chromosomes, some of which are derived from retrotransposons. Centromeric repeat sequences from Gong et al. [43] were aligned to v4.04 and v6.1 genomes with BLAST (v2.3.28 [50]) with alignments with greater than 99% identity over 95% of the query length retained. Expected centromere-specific repeats were identified in *Cen2*, *Cen5*, and *Cen7* in v.6.1 but not in v4.04 (Figure 3). In addition, the centromere-specific repeats were detected only in a single region in each respective chromosome in v.6.1. These results show significantly improved assembly of the centromeric sequences of v6.1 compared to v4.04. Two subtelomeric repeats have also been characterized in potato [53]. These two repeats were aligned to v6.1 and hits with greater than 90% identity over 80% of the query length were retained. We identified these repeats on 16 chromosomal ends in v.6.1 whereas 15 chromosomal ends were identified in v4.04 (Figure 3).

Annotation

To facilitate annotation of gene models, ONT cDNA sequences were generated from DM. DM was grown under a 16-hour day length in tissue culture and RNA was isolated from whole tissue-culture plants using a modified hot borate method [54]. DNA contaminants were removed using the Ambion Turbo DNase Kit (Thermofisher Scientific, Waltham, MA) and Dynabeads mRNA DIRECT Purification Kit (Thermofisher Scientific, Waltham, MA) was used to isolate mRNA. An ONT PCR-cDNA Sequencing library was constructed using the SQK-PCS109 kit (Oxford Nanopore, Oxford, UK) with the following modifications: input was increased to 5ng of mRNA, GC Melt Reagent (Takara Bio Inc., Kusatsu, Shiga, Japan) was included at a final concentration of 0.5M during reverse transcription and PCR, PrimeScript reverse transcriptase (Takara Bio Inc., Kusatsu, Shiga, Japan) was used for reverse transcription, 14 PCR cycles were performed with an extension time of 5 minutes, all Hula mixer steps were performed by hand, and the adapter ligation period was extended to 15 min with gentle mixing every five minutes. The completed library was sequenced using the MinION (MIN-101B) platform with a FLO-MIN106 Red D flow cell in two runs to maximize the yield of reads, the first connected to either an Apple Macintosh computer running MinKNOW v3.5.5 and the second connected to a ONT MinIT running MinKNOW v3.6.3 and MinIT 19.2.1. The sequenced ONT cDNA library was base called using Guppy (3.6.0+98ff765; [18]) on an Amazon Web Services p3.2xlarge NVIDIA Tesla V100 GPU instance with the parameters: --flowcell FLO-MIN106 --kit SQK-PCS109 -q 0 --qs.core_filtering --trim_strategy none --calib_detect. The reads that passed the basecaller quality filter were then processed with Pychopper (v.2.4.0; [55]) to identify full-length cDNA reads. The full-length and rescued cDNA reads were filtered with seqtk (seq -L 500; [19]) to remove reads less than 500 nt. The filtered cDNA reads were aligned to the genome assembly with minimap2 (v2.2.17) with the parameters (-a -x splice -uf -G 5000). The cDNA alignments were assembled using Stringtie2 (v2.1.2; [56]) (-L -m 500) and the assembled transcript sequences extracted with gffread (v0.11.7; [57]). Illumina TruSeq Stranded mRNA-Seq libraries previously prepared from DM leaf (NCBI Sequence Read Archive SRX2023785

and SRX2023786) and tuber (NCBI Sequence Read Archive SRX2023789 and SRX2023798) tissues were used to generate RNA-Seq transcript assemblies for gene model refinement. Reads were first cleaned using Cutadapt (v2.9; [26]) with the parameters: -n 2 -m 100 -q 10, aligned to the genome assembly using HISAT2 (v2.2.0; [58]) with the parameters: --max-intronlen 5000 --rna-strandness RF --no-unal --dta, and assembled using Stringtie (v2.1.1; [56]) with the parameter --rf and the assembled transcript sequences extracted with gffread (v0.11.7; [57]).

The BRAKER2 (git commit 6219573; [59]) gene prediction pipeline was used to train Augustus (v3.3.3;[60]) using GeneMark-ET (v4.57; [61]) and the RNA-Seq alignments to generate *ab initio* gene predictions. The BRAKER2 pipeline was run using the command line: braker.pl --species=DM_v6_1 --gff3 --softmasking --UTR=off --bam {RNA-seq.alns.bam}. *Ab initio* gene predictions were refined using PASA2 (v2.4.1; [62]) with the RNA-Seq and ONT cDNA transcript assemblies as evidence. Two rounds of annotation comparison were performed resulting in a set of 52,953 working gene models representing 40,652 loci. To identify high-confidence gene models, the working gene model set was searched against the PFAM database (v32;[63]) with hmmscan (HMMER v3.2.1; [64]) with a cutoff of --domE 1e-3 -E 1e-5 to identify gene models encoding a Pfam domain. Gene expression abundances (transcripts per million (TPM)) were generated using the leaf and tuber mRNAseq reads using Kallisto (v0.46.0; [65]).

High-confidence gene models were defined as having a TPM value > 0 in at least one RNA-Seq library and/or having a PFAM domain match. Gene models that were partial or had matches to transposable element-related PFAM domains were excluded from the high-confidence model set. A total of 32,917 loci encoding 44,851 gene models are contained within the high-confidence set (Table S7). To assign functional annotation to the gene models, searches using the predicted proteins were performed with the Arabidopsis proteome (TAIR10; [66]), the PFAM database (v32; [63]) ,and the Swiss-Prot plant proteins (release 2015_08). Search results were processed in the same order and

the function of the first hit encountered was assigned to the gene model. The quality of the annotation was evaluated using BUSCO [35] and both the working and high confidence gene sets in v6.1 provided excellent representation of the conserved orthologs with 93.5% complete in the working set and 93.0% complete in the high confidence set (Table S4). In contrast, the v4.04 annotation provided 74.6% complete BUSCO orthologs.

Conclusions

Using improved sequencing technologies, the genome sequence of the reference potato genotype DM was vastly improved in contiguity relative to the previous release, DM v4.04. Version 6.1 of the DM genome assembly represents 87.8% of the estimated genome with 595-fold increase in N50 contig size, 99% reduction in number of contigs and a 44-fold increase in N50 scaffold size. Importantly, 731.2 Mb of the 741.6 Mb assembly is non-gapped and anchored to the 12 chromosomes indicating a high degree of contiguity that was reflected in a 'reference quality' LAI score demonstrating the ability of advanced sequencing methods to assemble large contiguous regions of a medium-sized plant genome. With access to full-length cDNA sequences, 32,917 high-confidence protein-coding genes encoding 44,851 gene models were annotated which provided a substantial improvement in representation of conserved orthologs compared to the previous annotation that will facilitate future studies in potato biology, genetics and genomics.

Acknowledgements

This work was supported in part by funds from PepsiCo to CRB, Hatch funds to CRB (MICL02431), a USDA NIFA Predoctoral Fellowship (2017-67011-26038) awarded to GMP, and an NSF grant IOS-1444514 awarded to JJ and CRB. The authors acknowledge their colleague Mandy Waters from PepsiCo who aided in the organization of the project, Kayla Young from Phase Genomics who coordinated Hi-C sample preparation and sequencing,

and Shawn Sullivan from Phase Genomics for work on Hi-C scaffolding. The opinions in this study are those of the authors and do not necessarily represent the opinions or policies of PepsiCo Inc.

Availability of supporting information

The clone, DM1-3 516 R44, is available through the United States Department of Agriculture Potato Genebank via PI GS 233 (https://npgsweb.ars-grin.gov/gringlobal/accessiondetail.aspx?id=1812299). The raw genomic sequences and ONT cDNA are available in the NCBI Sequence Read Archive database under BioProject PRJNA636376. The genome assembly, annotation, CRL, and BUSCO results are available in GigaDB, Dryad Digital Repository (doi to be released upon publication), and on Spud DB (http://solanaceae.plantbiology.msu.edu/) via a JBrowse installation and download page.

Authors' contribution

CRB conceived the study. GMP, JPH, BV, and JCW performed the experiments. JB, JPH, JJ, GMP, SO, BV, JCW, and HZ analyzed data. CRB, JPH, JJ, GMP, BV, JCW, and HZ wrote the manuscript. All authors approved the final manuscript.

Competing interests

The authors declare no competing interests.

Abbreviations

BLAST: Basic Local Alignment Search Tool; BUSCO: Benchmarking Universal Single-Copy Orthologs; CRL: Custom Repeat Library; LAI: LTR Assembly Index; LTR: Long terminal repeat; NCBI: National Center for Biotechnology Information; nt: nucleotide; Oligo-FISH: Oligonucleotide fluorescent *in situ* hybridization; ONT: Oxford Nanopore Technologies; PGSC: Potato Genome Sequencing Consortium; RNA-Seq: RNA-Sequencing; TPM: Transcripts per million

Additional Files

Figure S1. Hi-C contact map showing the inter- and intra-chromosomal chromatin interactions in DM v6.1. Inter-chromosomal chromatin interactions are off the diagonal axis and intra-chromosomal chromatin interactions are within the blue boxes. Each pixel represents the degree of interaction between each 1 Mb locus, with a dark red color indicating a greater number of reads involved in the interaction.

Figure S2. Mapping of the DM x RH F1 population markers to the (a) DM v4.04 and the (b) DM v6.1 assembly. Flanking sequence (200 nt) of the markers was used for sequence alignments to the assembly using Vmatch [67]. The y-axis shows the map location in centimorgans and the x-axis shows the physical location in megabases.

Table S1. Sequence datasets used in this study. Total reads for Oxford Nanopore Technologies sequencing are passed reads after base calling.

Table S2. Oxford Nanopore Technologies whole-genome shotgun sequence reads used in the DM v6.1 assembly.

Table S3. Illumina whole-genome shotgun sequence read mapping statistics.

Table S4. Benchmarking Universal Single Copy Orthologs (BUSCO, [35]) results of the DM genome assemblies and annotation.

Table S5. Centromere positions in the DM v6.1 assembly.

Table S6. Repetitive sequence content in v4.04 and v6.1 DM 1-3 516 R44 genome assemblies.

Table S7. DM v6.1 gene annotation summary.

Figure legends

Figure 1. Doubled monoploid potato clone, DM1-3 516 R44. (a) Aboveground tissues and (b) tubers from the doubled monoploid potato clone, DM1-3 516 R44. Photos courtesy of Joseph Coombs.

Figure 2. Genome-wide LTR Assembly Index (LAI; [36]**) scores for DM assembly v.4.04 (V4) and v.6.1 (V6**). LAI was calculated for 3-Mb sliding windows with a 300-kb step size.

Figure 3. Distribution of subtelomeric repeat sequences, centromeric repeat sequences, CENH3 chromatin immunoprecipitation-sequencing (ChIP-seq) alignments, and oligonucleotide fluorescent *in situ* hybridization probes. (a) Distribution of features on DM v6.1 assembly. (b) Distribution of features on DM v4.04 assembly. Red and green rectangles represent the positions of the two "barcode" oligonucleotide fluorescent *in situ* hybridization probes [40]. For CENH3 ChIP-seq reads, chromosomes were divided into 100-kb windows and CENH3 read number in each window was calculated and plotted [43]. Circles represent centromeric repeats [43]. Triangles represent subtelomeric repeats [53].

Figure 4. Improved assembly of the centromeric regions in DM v6.1. (a) CENH3 read distribution on centromere 7. (b) CENH3 read distribution on centromere 10. Chromosomes were divided into 100 kb windows and the CENH3 ChIP-seq read number [43] in each window was calculated and plotted. Red circles represent centromeric repeats. Upper panel shows the CENH3 ChIP-seq read distribution in the DM v4.04 assembly, lower panel shows the distribution in the DM v6.1 assembly.

Figure 5. Whole-genome alignment of the DM v4.04 vs v6.1 DM genome assemblies. Whole-genome alignments of the long-read, chromosome scale DM v6.1 assembly with

the DM 4.04 genome assembly using D-GENIES [46] reveals concordance in the euchromatic arms but misassemblies in the pericentromeric regions.

Table 1. Assembly metrics of the DM 1-3 R44 v4 and v6 assemblies

	v4.03[a]	v4.04[b]	v6.1[c]
Total assembly size	773.0 Mb	884.1 Mb	741.6 Mb
Total non-gapped size	676.3 Mb	728.7 Mb	741.5 Mb
Contig N50 size	31,914 bp	29,071 bp	17,312,182 bp
Total contig number	60,068	170,833	1,382
Scaffold N50 size	1,344,915 bp	1,344,915 bp	59,670,755 bp
Scaffold number	14,853	14,853	288

[a] PGSC contigs and scaffolds downloaded from NCBI: AEWC01000001-AEWC01060068; JH137791-JH152643 [1, 2].

[b] DM v4.04 is composed of v4.03 plus an additional 110,765 unanchored contigs (55.7 Mb) [3].

[c] The DM v6.1 scaffolds are composed of the 12 chromosome-scale pseudomolecules and 276 unanchored scaffolds.

Table 2. Chromosome lengths and gap (N) content in DM v4.04 and v6.1.

	DM v4.04					DM v6.1				
Chromosome	Total Chromosome Length (bp)	Total Sequence Length (bp)	% Sequence	Total Gap Length (bp)	% Gaps	Total Chromosome Length (bp)	Total Sequence Length (bp)	% Sequence	Total Gap Length (bp)	% Gaps
chr01	88,663,952	77,894,594	87.85%	10,769,358	12.15%	88,591,686	88,579,186	99.99%	12,500	0.01%
chr02	48,614,681	42,696,816	87.83%	5,917,865	12.17%	46,102,915	46,100,415	99.99%	2,500	0.01%
chr03	62,290,286	53,928,846	86.58%	8,361,440	13.42%	60,707,570	60,704,570	100.00%	3,000	0.00%
chr04	72,208,621	62,203,573	86.14%	10,005,048	13.86%	69,236,331	69,230,831	99.99%	5,500	0.01%
chr05	52,070,158	46,610,373	89.51%	5,459,785	10.49%	55,599,697	55,591,197	99.98%	8,500	0.02%
chr06	59,532,096	51,644,783	86.75%	7,887,313	13.25%	59,091,578	59,085,578	99.99%	6,000	0.01%
chr07	56,760,843	49,550,308	87.30%	7,210,535	12.70%	57,639,317	57,635,317	99.99%	4,000	0.01%
chr08	56,938,457	49,300,183	86.59%	7,638,274	13.41%	59,226,000	59,217,000	99.98%	9,000	0.02%
chr09	61,540,751	53,891,571	87.57%	7,649,180	12.43%	67,600,300	67,594,300	99.99%	6,000	0.01%
chr10	59,756,223	52,349,496	87.61%	7,406,727	12.39%	61,044,151	61,037,651	99.99%	6,500	0.01%
chr11	45,475,667	40,128,174	88.24%	5,347,493	11.76%	46,777,387	46,772,387	99.99%	5,000	0.01%
chr12	61,165,649	53,902,062	88.12%	7,263,587	11.88%	59,670,755	59,658,755	99.98%	12,000	0.02%
Total Pseudomolecules	725,017,384	634,100,779	87.46%	90,916,605	12.54%	731,287,687	731,207,187	99.99%	80,500	0.01%
Unanchored Sequences	159,090,912	94,595,563	59.46%	64,495,349	40.54%	10,297,348	10,289,348	99.92%	8,000	0.08%
Total Assembly	884,108,296	728,696,342	82.42%	155,411,954	17.58%	741,585,035	741,496,535	99.99%	88,500	0.01%

REFERENCES

1. The Potato Genome Sequencing Consortium. Genome sequence and analysis of the tuber crop potato. Nature. 2011;475 7355:189-95. doi:10.1038/nature10158.
2. Sharma SK, Bolser D, de Boer J, Sonderkaer M, Amoros W, Carboni MF, et al. Construction of reference chromosome-scale pseudomolecules for potato: integrating the potato genome with genetic and physical maps. G3 (Bethesda). 2013;3 11:2031-47. doi:10.1534/g3.113.007153.
3. Hardigan MA, Crisovan E, Hamilton JP, Kim J, Laimbeer P, Leisner CP, et al. Genome Reduction Uncovers a Large Dispensable Genome and Adaptive Role for Copy Number Variation in Asexually Propagated *Solanum tuberosum*. Plant Cell. 2016;28 2:388-405. doi:10.1105/tpc.15.00538.
4. Kloosterman B, Abelenda JA, Gomez Mdel M, Oortwijn M, de Boer JM, Kowitwanich K, et al. Naturally occurring allele diversity allows potato cultivation in northern latitudes. Nature. 2013;495 7440:246-50. doi:10.1038/nature11912.
5. Uitdewilligen JG, Wolters AM, D'Hoop B B, Borm TJ, Visser RG and van Eck HJ. A next-generation sequencing method for genotyping-by-sequencing of highly heterozygous autotetraploid potato. PloS ONE. 2013;8 5:e62355. doi:10.1371/journal.pone.0062355.
6. Manrique-Carpintero NC, Coombs JJ, Pham GM, Laimbeer FPE, Braz GT, Jiang J, et al. Genome Reduction in Tetraploid Potato Reveals Genetic Load, Haplotype Variation, and Loci Associated With Agronomic Traits. Front Plant Sci. 2018;9:944. doi:10.3389/fpls.2018.00944.
7. Witek K, Jupe F, Witek AI, Baker D, Clark MD and Jones JDG. Accelerated cloning of a potato late blight-resistance gene using RenSeq and SMRT sequencing. Nat Biotech. 2016;34:656-60. doi:10.1038/nbt.3540
8. Hardigan MA, Laimbeer FPE, Newton L, Crisovan E, Hamilton JP, Vaillancourt B, et al. Genome diversity of tuber-bearing *Solanum* uncovers complex evolutionary history and targets of domestication in the cultivated potato. Proc Natl Acad Sci U S A. 2017;114 46:E9999-E10008. doi:10.1073/pnas.1714380114.
9. Manrique-Carpintero NC, Coombs JJ, Veilleux RE, Buell CR and Douches DS. Comparative Analysis of Regions with Distorted Segregation in Three Diploid Populations of Potato. G3 (Bethesda). 2016;6 8:2617-28. doi:10.1534/g3.116.030031.
10. Pham GM, Newton, L., Wiegert-Rininger, K., Vaillancourt, B., Douches, D.S., Buell, C.R. . Extensive genome heterogeneity leads to preferential allele expression and copy number-dependent expression in cultivated potato. The Plant Journal. 2017;92:624-37.
11. Pham GM, Braz, G.T., Conway, M., Crisovan, E., Hamilton, J.P., Laimbeer, F.P.E., Manrique-Carpintero, N., Newton, L., Douches, D.S., Jiang, J., Veilleux, R.E., Buell, C.R. Genome-wide Inference of Somatic Translocation Events During Potato Dihaploid Production. The Plant Genome. 2019;12 doi:10.3835/plantgenome2018.10.0079.

12. Kyriakidou M, Anglin NL, Ellis D, Tai HH and Stromvik MV. Genome assembly of six polyploid potato genomes. Sci Data. 2020;7 1:88. doi:10.1038/s41597-020-0428-4.
13. Zeng ZX, Zhang, W.L., Marand, A.P., Zhu, B., Buell, C.R., Jiang, J.M. . Cold stress of plant tissues induces enhanced chromatin accessibility in genic regions marked by bivalent histone modifications H3K4me3 and H3K27me3. Genome Biology. 2019;20 123 doi:https://doi.org/10.1186/s13059-019-1731-2.
14. Jiao WB and Schneeberger K. The impact of third generation genomic technologies on plant genome assembly. Curr Opin Plant Biol. 2017;36:64-70. doi:10.1016/j.pbi.2017.02.002.
15. Workman R, Fedak, R., Kilburn, D., Hao, S., Liu, K., Timp, W. High Molecular Weight DNA Extraction from Recalcitrant Plant Species for Third Generation Sequencing Nature Protocol Exchange. 2018; doi:doi:10.1038/protex.2018.059.
16. Doyle JJ, Doyle, J.L. . A rapid DNA isolation procedure for small quantities of fresh leaf tissue. Phytochemical Bulletin. 1987;19:11-5.
17. Burton JN, Adey A, Patwardhan RP, Qiu R, Kitzman JO and Shendure J. Chromosome-scale scaffolding of *de novo* genome assemblies based on chromatin interactions. Nat Biotechnol. 2013;31 12:1119-25. doi:10.1038/nbt.2727.
18. Guppy. https://community.nanoporetech.com. Accessed May 2020.
19. seqtk:Toolkit for processing sequences in FASTA/Q formats. https://github.com/lh3/seqtk. Accessed May 2020.
20. Kolmogorov M, Yuan J, Lin Y and Pevzner PA. Assembly of long, error-prone reads using repeat graphs. Nat Biotechnol. 2019;37 5:540-6. doi:10.1038/s41587-019-0072-8.
21. Vaser R, Sovic I, Nagarajan N and Sikic M. Fast and accurate *de novo* genome assembly from long uncorrected reads. Genome Res. 2017;27 5:737-46. doi:10.1101/gr.214270.116.
22. Li H. Minimap2: pairwise alignment for nucleotide sequences. Bioinformatics. 2018;34 18:3094-100. doi:10.1093/bioinformatics/bty191.
23. Loman NJ, Quick J and Simpson JT. A complete bacterial genome assembled *de novo* using only nanopore sequencing data. Nat Methods. 2015;12 8:733-5. doi:10.1038/nmeth.3444.
24. Li H, Handsaker B, Wysoker A, Fennell T, Ruan J, Homer N, et al. The Sequence Alignment/Map format and SAMtools. Bioinformatics. 2009;25 16:2078-9. doi.org/10.1093/bioinformatics/btp352.
25. Walker BJ, Abeel T, Shea T, Priest M, Abouelliel A, Sakthikumar S, et al. Pilon: an integrated tool for comprehensive microbial variant detection and genome assembly improvement. PLoS ONE. 2014;9 11:e112963. doi:10.1371/journal.pone.0112963.
26. Martin M. Cutadapt removes adapter sequences from high-throughput sequencing reads. EMBnetjournal. 2011;17 1 doi:http://dx.doi.org/10.14806/ej.17.1.200.

27. Li H. Aligning sequence reads, clone sequences and assembly contigs with BWA-MEM. arXiv. 2013;1303.3997v2.
28. Picard Tools. https://broadinstitute.github.io/picard/. Accessed May 2020.
29. Durand NC, Shamim MS, Machol I, Rao SS, Huntley MH, Lander ES, et al. Juicer Provides a One-Click System for Analyzing Loop-Resolution Hi-C Experiments. Cell Syst. 2016;3 1:95-8. doi:10.1016/j.cels.2016.07.002.
30. Dudchenko O, Batra SS, Omer AD, Nyquist SK, Hoeger M, Durand NC, et al. *De novo* assembly of the *Aedes aegypti* genome using Hi-C yields chromosome-length scaffolds. Science. 2017;356 6333:92-5. doi:10.1126/science.aal3327.
31. Durand NC, Robinson JT, Shamim MS, Machol I, Mesirov JP, Lander ES, et al. Juicebox Provides a Visualization System for Hi-C Contact Maps with Unlimited Zoom. Cell Syst. 2016;3 1:99-101. doi:10.1016/j.cels.2015.07.012.
32. NCBI Resource Coordinators. Database resources of the National Center for Biotechnology Information. Nucleic Acids Res. 2018;46 D1:D8-D13. doi:10.1093/nar/gkx1095.
33. Kim D, Song L, Breitwieser FP and Salzberg SL. Centrifuge: rapid and sensitive classification of metagenomic sequences. Genome Res. 2016;26 12:1721-9. doi:10.1101/gr.210641.116.
34. Altschul SF, Gish W, Miller W, Meyers EW and Lipman DJ. Basic Local Alignment Search Tool. J Mol Biol. 1990;215:403-10.
35. Simao FA, Waterhouse RM, Ioannidis P, Kriventseva EV and Zdobnov EM. BUSCO: assessing genome assembly and annotation completeness with single-copy orthologs. Bioinformatics. 2015;31 19:3210-2. doi:10.1093/bioinformatics/btv351.
36. Ou SJ, Chen JF and Jiang N. Assessing genome assembly quality using the LTR Assembly Index (LAI). Nucleic Acids Research. 2018;46 21. doi:10.1093/nar/gky730.
37. Ellinghaus D, Kurtz S and Willhoeft U. LTRharvest, an efficient and flexible software for de novo detection of LTR retrotransposons. BMC Bioinformatics. 2008;9:18. doi:10.1186/1471-2105-9-18.
38. Ou S and Jiang N. LTR_FINDER_parallel: parallelization of LTR_FINDER enabling rapid identification of long terminal repeat retrotransposons. Mobile DNA. 2019;10:48. doi:10.1186/s13100-019-0193-0.
39. Ou S and Jiang N. LTR_retriever: A Highly Accurate and Sensitive Program for Identification of Long Terminal Repeat Retrotransposons. Plant Physiol. 2018;176 2:1410-22. doi:10.1104/pp.17.01310.
40. Braz GT, He L, Zhao H, Zhang T, Semrau K, Rouillard JM, et al. Comparative oligo-FISH Mapping: An Efficient and Powerful Methodology to Reveal Karyotypic and Chromosomal Evolution. Genetics. 2018;208:513-23. doi:10.1534/genetics.117.300344.
41. Manrique-Carpintero NC, Coombs JJ, Cui Y, Veilleux RE, Buell CR and Douches D. Genetic map and quantitative trait locus analysis of agronomic traits in a diploid potato population using single nucleotide polymorphism markers. Crop Science. 2015;55:2566-79.

42. VMATCH: The Vmatch large scale sequence analysis software. http://www.vmatch.de/ Accessed May 2020.
43. Gong Z, Wu Y, Koblizkova A, Torres GA, Wang K, Iovene M, et al. Repeatless and repeat-based centromeres in potato: implications for centromere evolution. Plant Cell. 2012;24 9:3559-74. doi:10.1105/tpc.112.100511.
44. Quinlan AR and Hall IM. BEDTools: a flexible suite of utilities for comparing genomic features. Bioinformatics. 2010;26 6:841-2. doi:Doi 10.1093/Bioinformatics/Btq033.
45. Ou S, Liu J, Chougule KM, Fungtammasan A, Seetharam AS, Stein JC, et al. Effect of sequence depth and length in long-read assembly of the maize inbred NC358. Nature Communications. 2020;11 1:2288. doi:10.1038/s41467-020-16037-7.
46. Cabanettes F and Klopp C. D-GENIES: dot plot large genomes in an interactive, efficient and simple way. PeerJ. 2018;6:e4958. doi:10.7717/peerj.4958.
47. Flynn JM, Hubley R, Goubert C, Rosen J, Clark AG, Feschotte C, et al. RepeatModeler2 for automated genomic discovery of transposable element families. Proc Natl Acad Sci U S A. 2020;117 17:9451-7. doi:10.1073/pnas.1921046117.
48. Campbell MS, Law M, Holt C, Stein JC, Moghe GD, Hufnagel DE, et al. MAKER-P: a tool kit for the rapid creation, management, and quality control of plant genome annotations. Plant Physiol. 2014;164 2:513-24. doi:10.1104/pp.113.230144.
49. Plant Protein Database. http://www.hrt.msu.edu/uploads/535/78637/alluniRefprexp070416.gz. Accessed April 28, 2020.
50. Camacho C, Coulouris G, Avagyan V, Ma N, Papadopoulos J, Bealer K, et al. BLAST+: architecture and applications. BMC Bioinformatics. 2009;10:421. doi:10.1186/1471-2105-10-421.
51. Jurka J, Kapitonov VV, Pavlicek A, Klonowski P, Kohany O and Walichiewicz J. Repbase Update, a database of eukaryotic repetitive elements. Cytogenet Genome Res. 2005;110 1-4:462-7.
52. Chen N. Using RepeatMasker to identify repetitive elements in genomic sequences. Curr Protoc Bioinformatics. 2004;Chapter 4:Unit 4 10.
53. Torres GA, Gong, Z., Iovene, M., Cory D. Hirsch, C.D., Buell, C.R., Bryan, G.J., Petr Novák, P., Jiří Macas, J., Jiang, J. . Organization and evolution of subtelomeric DNA in the potato genome. Genes, Genomes, and Genetics. 2011;1:85-92.
54. Wan CY and Wilkins TA. A modified hot borate method significantly enhances the yield of high-quality RNA from cotton (*Gossypium hirsutum* L.). Anal Biochem. 1994;223 1:7-12. doi:10.1006/abio.1994.1538.
55. Pychopper: A tool to identify, orient, trim, and rescue full length cDNA reads. https://github.com/nanoporetech/pychopper. Accessed May 2020.
56. Kovaka S, Zimin AV, Pertea GM, Razaghi R, Salzberg SL and Pertea M. Transcriptome assembly from long-read RNA-seq alignments with StringTie2. Genome Biol. 2019;20 1:278. doi:10.1186/s13059-019-1910-1.

57. Pertea G, Pertea, M. . GFF Utilities: GffRead and GffCompare [version 1; peer review: 2 approved]. F1000Research. 2020;9:304. doi:https://doi.org/10.12688/f1000research.23297.1.
58. Kim D, Paggi JM, Park C, Bennett C and Salzberg SL. Graph-based genome alignment and genotyping with HISAT2 and HISAT-genotype. Nat Biotechnol. 2019;37 8:907-15. doi:10.1038/s41587-019-0201-4.
59. Hoff KJ, Lomsadze A, Borodovsky M and Stanke M. Whole-Genome Annotation with BRAKER. Methods Mol Biol. 2019;1962:65-95. doi:10.1007/978-1-4939-9173-0_5.
60. Stanke M, Schoffmann O, Morgenstern B and Waack S. Gene prediction in eukaryotes with a generalized hidden Markov model that uses hints from external sources. BMC Bioinformatics. 2006;7:62.
61. Lomsadze A, Burns PD and Borodovsky M. Integration of mapped RNA-Seq reads into automatic training of eukaryotic gene finding algorithm. Nucleic Acids Res. 2014;42 15:e119. doi:10.1093/nar/gku557.
62. Haas BJ, Delcher AL, Mount SM, Wortman JR, Smith RK, Jr., Hannick LI, et al. Improving the Arabidopsis genome annotation using maximal transcript alignment assemblies. Nucleic Acids Res. 2003;31 19:5654-66.
63. Finn RD, Mistry J, Tate J, Coggill P, Heger A, Pollington JE, et al. The Pfam protein families database. Nucleic Acids Res. 2010;38 Database issue:D211-22. ddoi: 10.1093/nar/gkp985.
64. Eddy SR. Accelerated Profile HMM Searches. PLoS Comput Biol. 2011;7 10:e1002195. doi:10.1371/journal.pcbi.1002195.
65. Bray NL, Pimentel H, Melsted P and Pachter L. Near-optimal probabilistic RNA-seq quantification. Nat Biotechnol. 2016;34 5:525-7. doi:10.1038/nbt.3519.
66. Lamesch P, Berardini TZ, Li D, Swarbreck D, Wilks C, Sasidharan R, et al. The Arabidopsis Information Resource (TAIR): improved gene annotation and new tools. Nucleic Acids Res. 2012;40 Database issue:D1202-10. doi:10.1093/nar/gkr1090.
67. Abouelhoda MI, Kurtz S and Ohlebusch E. Replacing suffix trees with enhanced suffix arrays. Journal of Discrete Algorithms. 2004;2 1:53-86. doi:https://doi.org/10.1016/S1570-8667(03)00065-0.

Figure 1

Click here to download Figure Fig_1.pptx

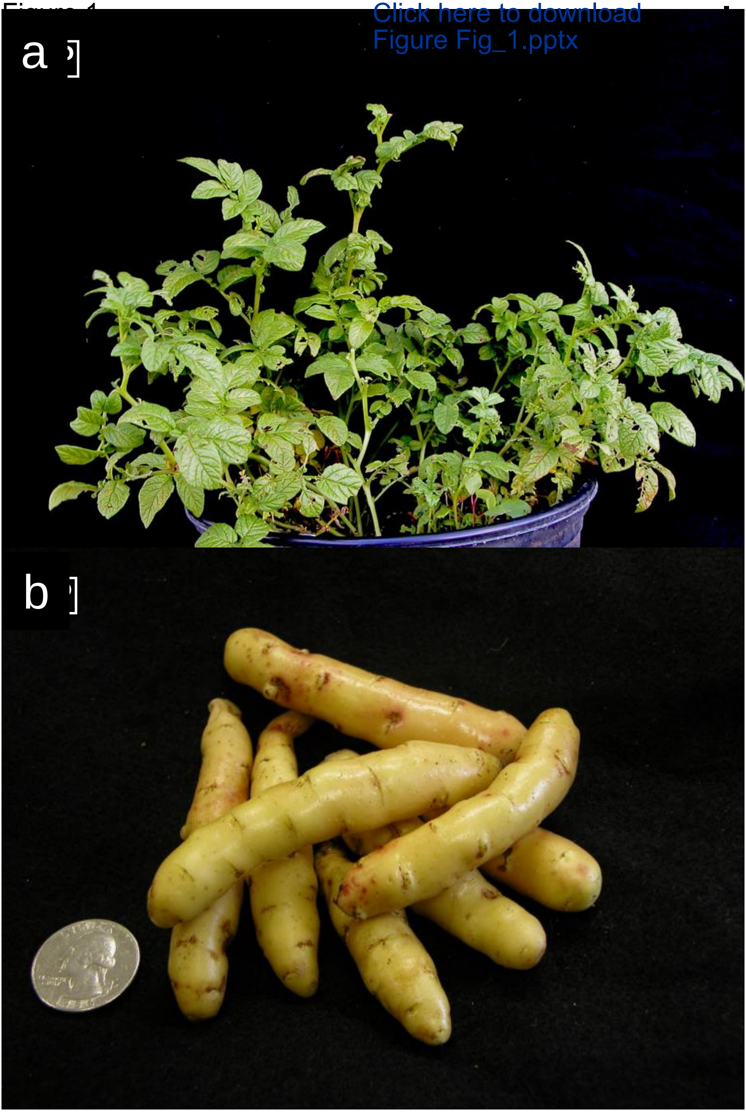

Figure 2

Click here to download Figure Fig_2.pptx

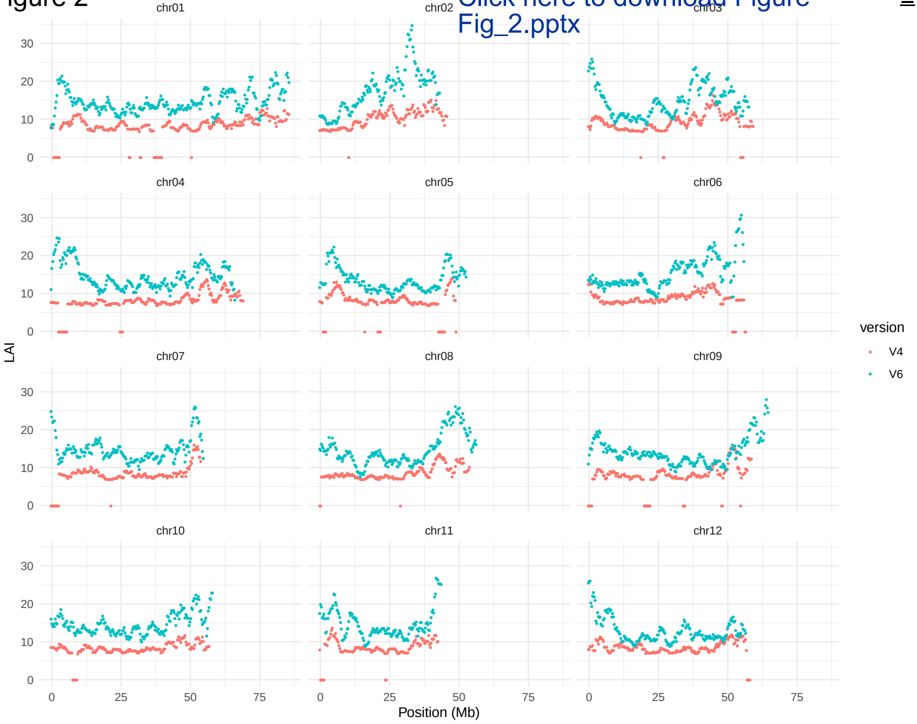

Figure 3

Click here to download Figure Fig_3.pptx

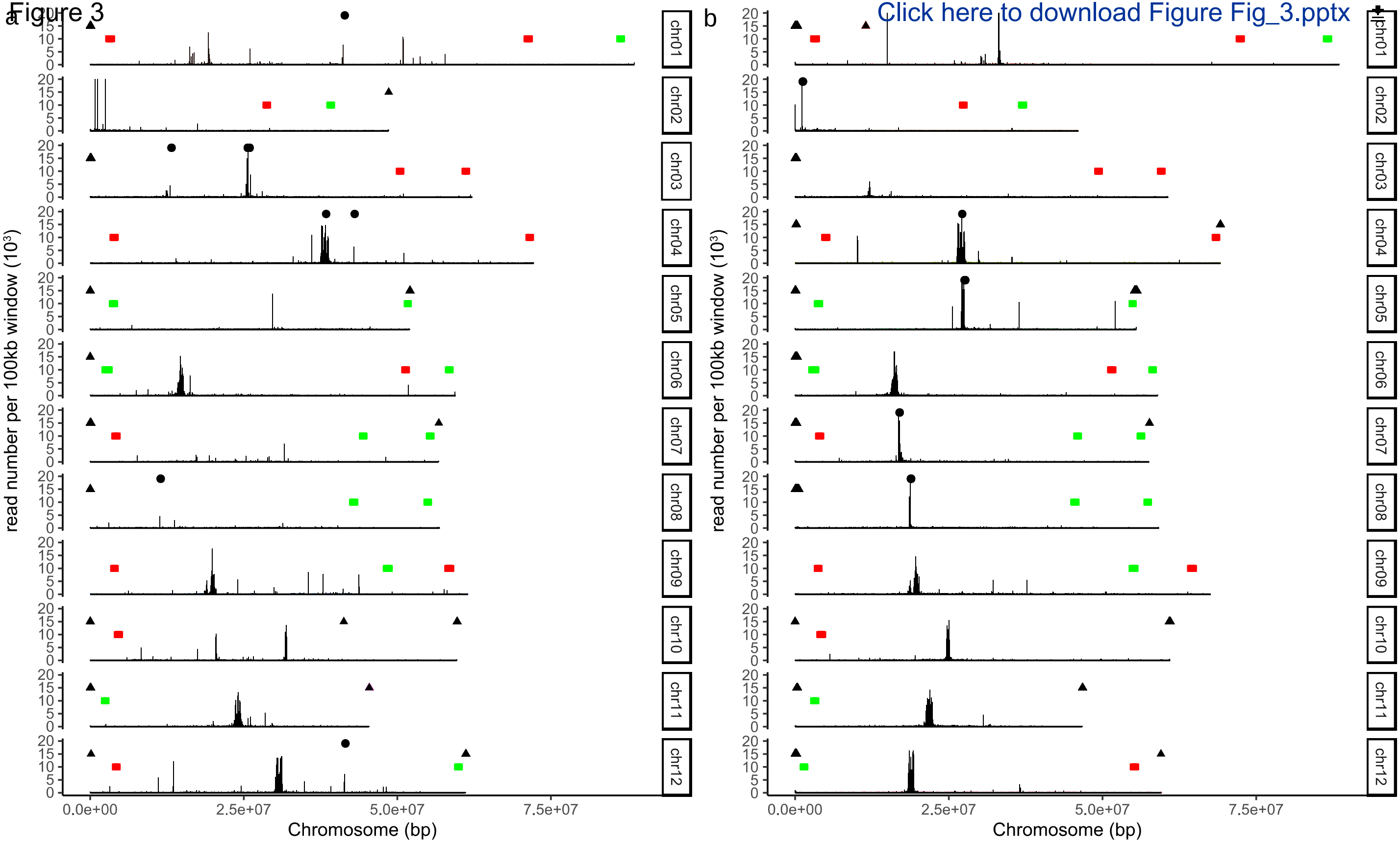

Figure 4

Click here to download Figure Fig_4.pptx

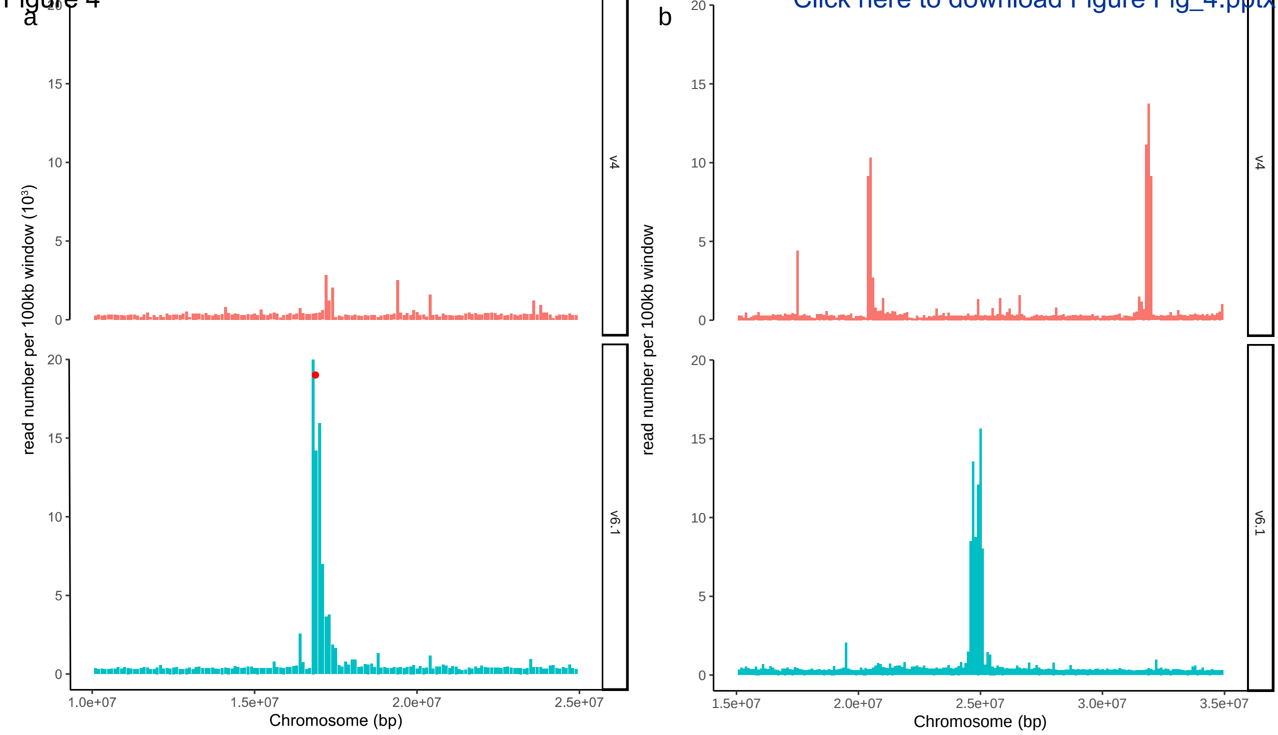

Figure 5 Click here to download Figure Fig_5.pptx

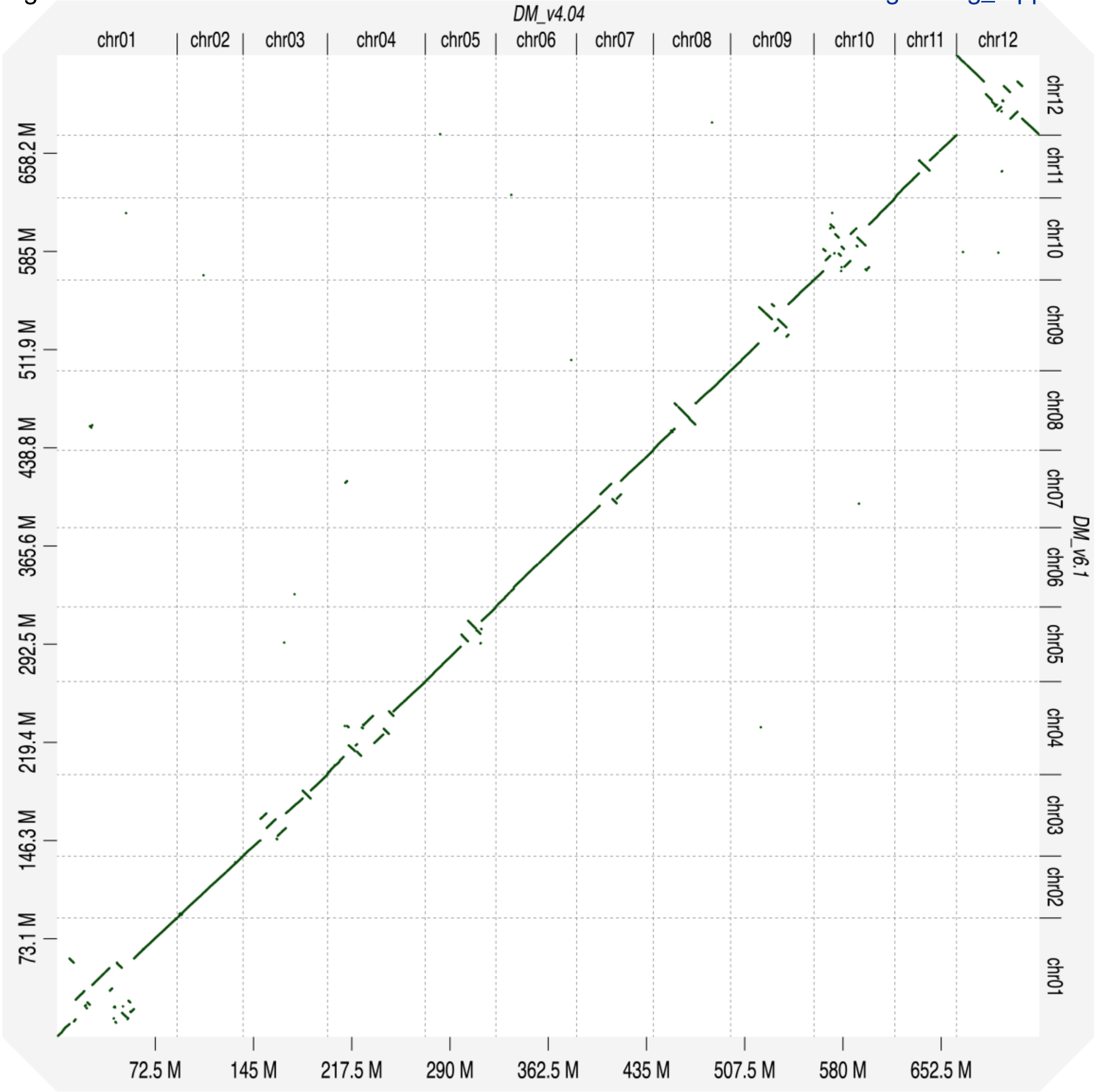

Figure S1

Click here to download Figure Fig_S1.pptx

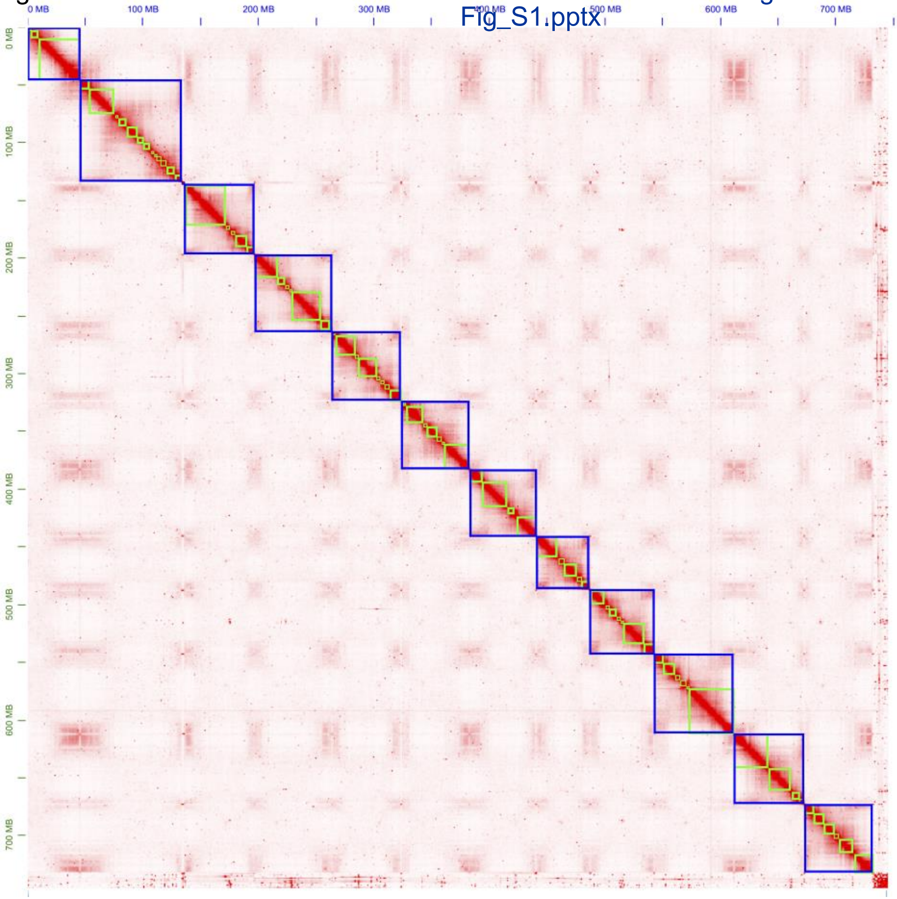

Figure S2

Click here to download Figure Fig_S2.pptx

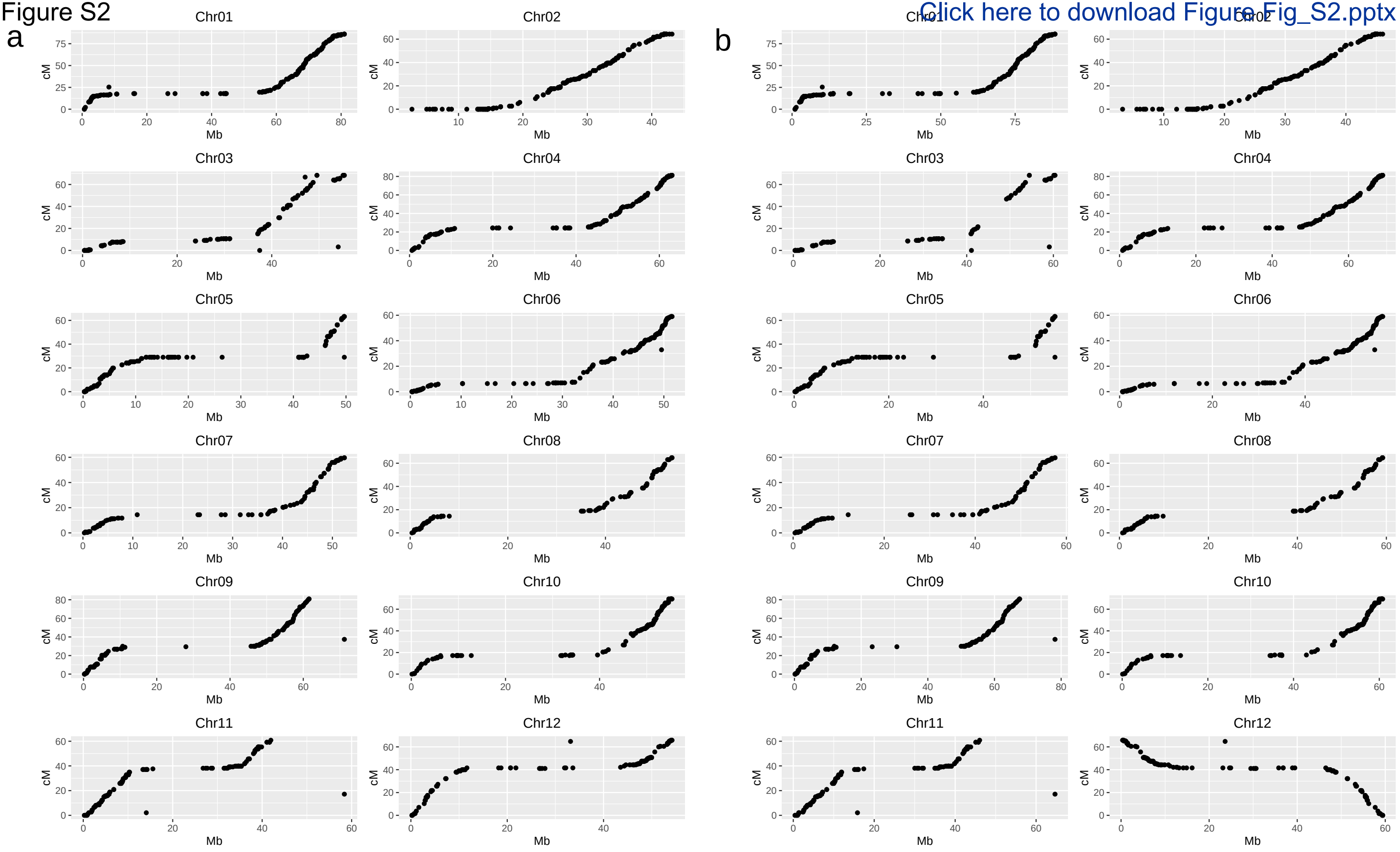

Table S1

Click here to access/download
Supplementary Material
Table_S1.xlsx

Table S2

Click here to access/download
Supplementary Material
Table_S2.xlsx

Table S3

Click here to access/download
Supplementary Material
Table_S3.xlsx

Table S4

Click here to access/download
Supplementary Material
Table_S4.xlsx

Table S5

Click here to access/download
Supplementary Material
Table_S5.xlsx

Table S6

Click here to access/download
Supplementary Material
Table_S6.xlsx

Table S7

Click here to access/download
Supplementary Material
Table_S7.xlsx
