# Supplementary figures and images for: Construction of a chromosome-scale long-read reference genome assembly for potato
Source: Gigascience. 2020 Sep 23;9(9):giaa100. doi: 10.1093/gigascience/giaa100 (PMC7509475; doi:10.1093/gigascience/giaa100)

## Slide 1
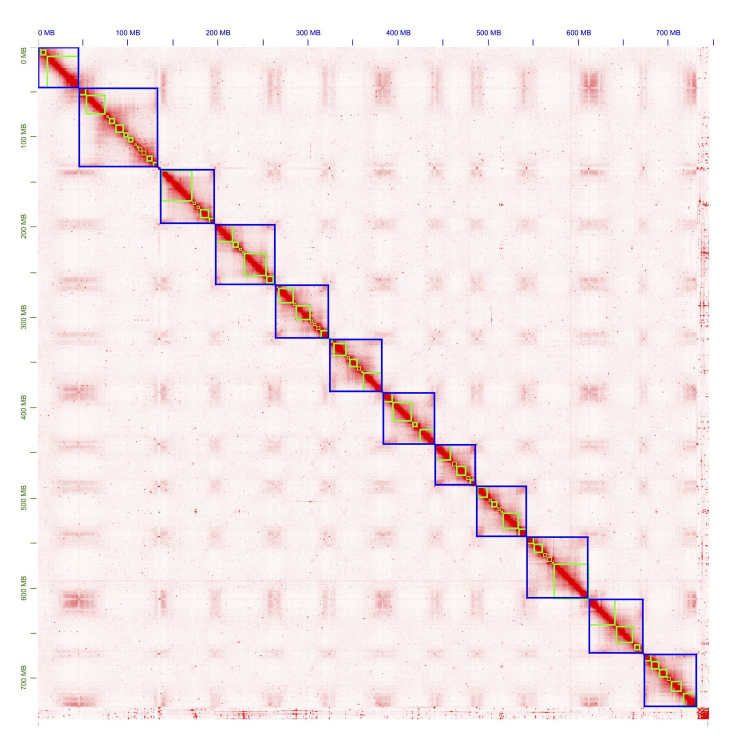

Supplement: giaa100_Supplemental_Files [file giaa100_supplemental_files.zip › Fig_S1.pptx]

## Slide 1
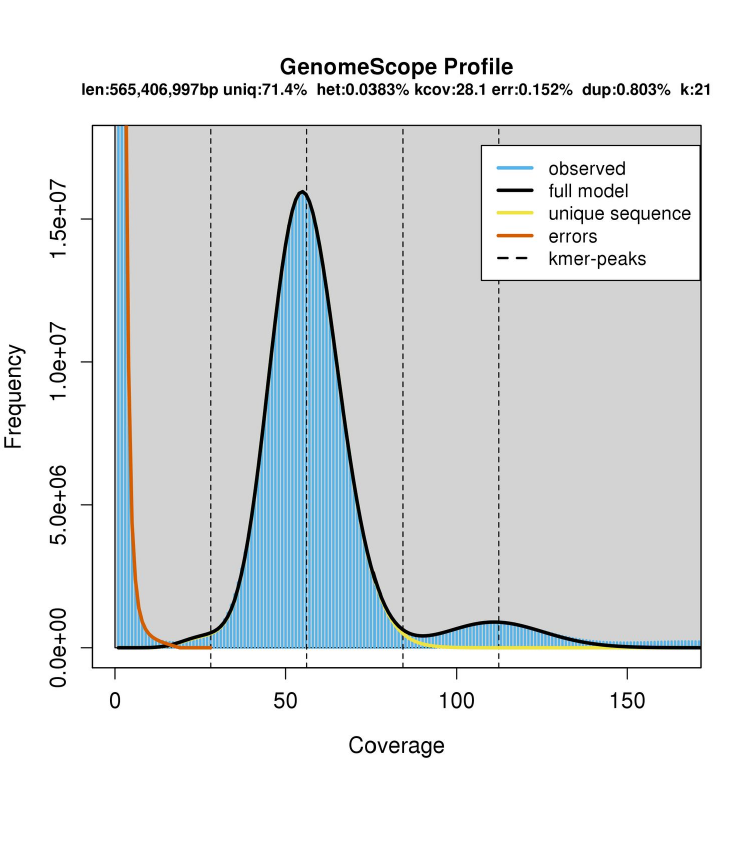

Supplement: giaa100_Supplemental_Files [file giaa100_supplemental_files.zip › Fig_S2.pptx]

## Slide 1
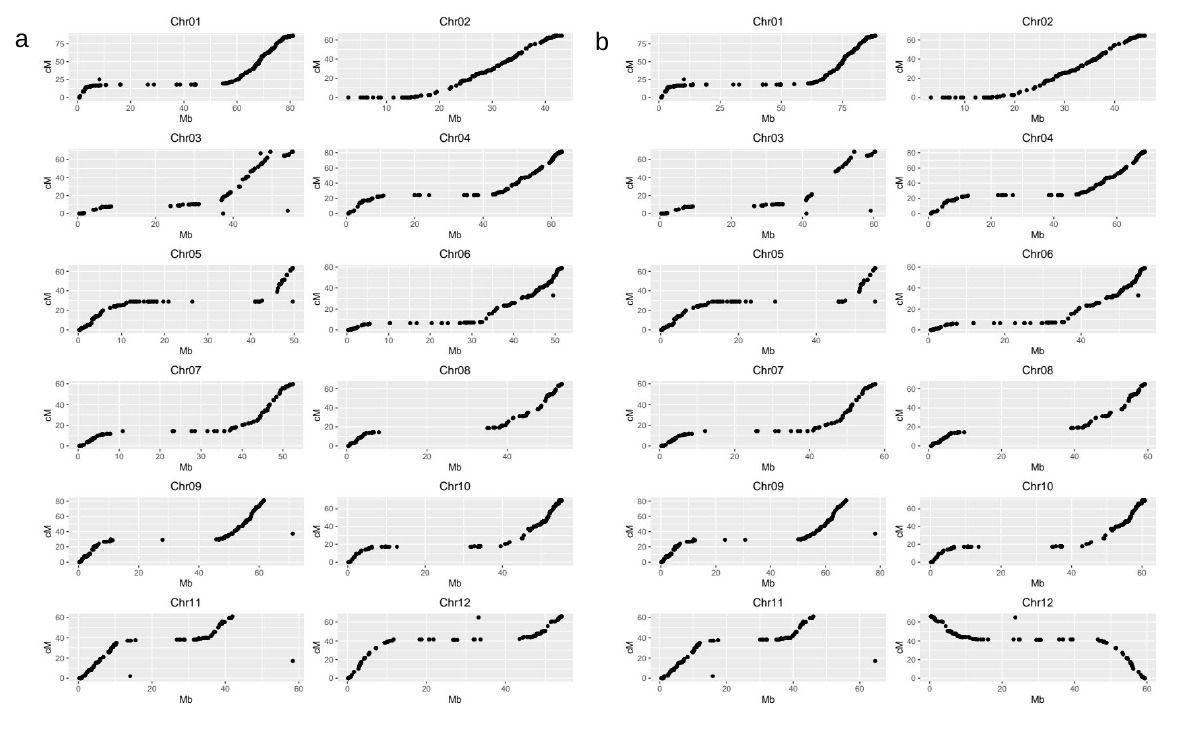

a
b

Supplement: giaa100_Supplemental_Files [file giaa100_supplemental_files.zip › Fig_S3.pptx]
