# Supplementary material for: Construction of a chromosome-scale long-read reference genome assembly for potato
Source: Gigascience. 2020 Sep 23;9(9):giaa100. doi: 10.1093/gigascience/giaa100 (PMC7509475; doi:10.1093/gigascience/giaa100)
Supplement: giaa100_Response_to_Reviewer_Comments_Original_Submission [file giaa100_response_to_reviewer_comments_original_submission.pdf]

Author's Response To Reviewer Comments

Close

Dear Zhou,

We have revised our manuscript to address the reviewer's comments and provided (below) a point-by-point response to t RRIDs and made a few other minor edits to the manuscript. We have uploaded a marked up copy of the revised manuscri to the GigaScience website. We have released our files on the Dryad Digital Repository and in the NCBI SRA as well. We h publication in GigaScience.

C. Robin Buell

Response to Reviewer's Comments

Reviewer reports:
Reviewer #1: Review: Construction of a chromosome-scale long-read reference genome assembly for potato

The authors described the updated genome assembly for potato and provided the genome annotations, especially the ann genome assembly represented much improvement over the released ones. This study and the associated data are very m breeding communities.

While the manuscript is well written, we have a few minor comments:

1. First of all, the main text has no line numbers for reviewers. It is a little bit hard to input specific comments.

Author Response: We have inserted line numbers in the revised document.

2. Would the authors like to report the ONT sequencing in depth both the main text and Tables, like Table S2?

Author Response: The coverage of the reads used in the assembly has been added to the main text and Table S2.

3. For each polishing steps, would you like to report the improvement (or changes) you gained. Also, I may want to know polishment? Why not 2 or 4 rounds?

Author Response: Polishing is a tradeoff between fixing true assembly errors and polishing errors into the assembly, espe the risk of degrading repetitive regions while fixing few true errors. At the third round of pilon we hit a plateau of errors fi feel the final BUSCO metrics for the genome assembly and annotation and the genome assembly LAI score show the polis

4. Do you have any means to examine whether there are redundances of haplotigs? Do you think Redundans (https://urldefense.com/v3/__https://github.com/lpryszcz/redundans__;!!HXCxUKc!i8YGoP6U4aW8ImOHsSvIcjQQv1Lfhs could help in this?

Author Response: As the DM potato is a doubled monoploid propagated by cloning, it is homozygous albeit mutation could have performed a GenomeScope analysis using the Illumina whole genome shotgun sequencing reads. We have added the the genome assessment section and added a new supplemental Figure (Figure S2) showing the kmer distribution generat DM to be homozygous so the presence of haplotigs is not expected.

5. In the genome assessment section, would you like to report the heterozygosites (polymorphic sites) you called from sh for readers.

Author Response: See response to #4 above.

Reviewer #2: Pham et al. present a reference-quality genome assembly for a doubled monoploid potato clone using Oxfor scaffolding. Previously generated resources, including a genetic map from 190 individuals, were used to validate the place pseudomolecules. New Oxford Nanopore cDNAs and published RNA-seq libraries were used to annotate gene models, whic ~93% of the BUSCO orthologs.

This new cultivated potato assembly is a considerable improvement over previous versions and will be a welcome addition
plant genome assemblies. Overall, the manuscript is well written and organized with adequate detail to reproduce the ass
of analysis here is probably more than sufficient for a data note, and all figures, tables, and supplementary materials are

I have some minor comments on a few places where I feel additional details or clarification would be helpful:

1. Was the average size of size of the isolated high molecular weight DNA measured?

Author Response: Based on the Fragment Analyzer results, we estimate the size of the high molecular weight DNA as > 6

2. Please specify the pore type in the flow cell.

Author Response: We have updated the text to reflect which pore type were in the flow cells used in the study.

3. What was the average quality for the ONT reads? I am not very familiar with Guppy, but can the --qscore_filtering opti
min_qscore ? If so, what is the default minimum Q Guppy uses?

Author Response: The mean quality score for all the reads is 10.2. The median quality score for the reads that passed the

Using the qscore_filtering option turns on the binning of the base called reads into pass or fail bins based on the min_qsc
set by the configuration profile provided with guppy based on the flowcell and kit parameters provided. It should be noted
steps are tuned to handle the Nanopore error profile.

4. Why was Flye chosen over other assemblers?

Author Response: Flye was published in 2019 in Nature Biotechnology as we started this work. Our own testing at the tim
results with our nanopore data in line with the results shown in the paper and on the GitHub repo for other organisms. Fly
and maintained with regular updates.

5. Why was the consensus VCF generated prior to Pilon polishing?

Author Response: The consensus VCF is generated by the nanopolish variants subcommand in order to update the assemb
vcf2fasta subcommand.

This is described in our methods for the polishing: "An updated consensus VCF file was generated using nanopolish varian
assembly generated using the VCF file with nanopolish vcf2fasta."

6. How many Illumina reads were used with Pilon? The ~459 million mentioned in the contiguity and accuracy section?

Author Response: Correct, we have updated the text to specify the Illumina library id (PEP_AA_01) in the section describi
accuracy section. Also, Table S1 was reporting read pair count for the Illumina libraries and this has been updated to show

7. Could you elaborate on how "recombination bins [were] manually adjusted to eliminate incorrect bins"? How were these

Author Response: There are occasionally mistakes in the genotyping data of one or two individuals in the population that
recombination events in the genetic map. This is highly unlikely in one individual and these positions were rescored as 'no

8. Intact LTRs were annotated using LTRharvest, LTR_finder and LTR_retriever for assessing assembly continuity using the
later used in RepeatModeler to mask the assembly or included in the final custom repeat library? Which set(s) of repeats w
to gene prediction?

Author Response: The annotation of the genome assembly and the genome LAI analysis were performed independently.

The construction of the custom repeat library, repeat masking, and the use of the repeat masked genomes in the annotati
commands used, are fully described in the methods.

9. How much cDNA and RNA-Seq transcript data were ultimately aligned to the genome and used for gene annotation?

Author Response: We have added the alignment rates for the nanopore and the RNA-seq data to the text.

10. What do the green boxes in Fig S1 represent?

Author Response: The green boxes are the individual scaffolds within the pseudomolecule. The boundaries of the pseudom
We have added text to the legend of Figure S1 to clarify what the blue and green boxes represent.

Close
