# Supplementary material for: Construction of a chromosome-scale long-read reference genome assembly for potato
Source: Gigascience. 2020 Sep 23;9(9):giaa100. doi: 10.1093/gigascience/giaa100 (PMC7509475; doi:10.1093/gigascience/giaa100)
Supplement: giaa100_Reviewer_1_Report_Original_Submission — Jian-Feng Mao, Ph.D. -- 7/7/2020 Reviewed [file giaa100_reviewer_1_report_original_submission.pdf]

Reviewer Report

Title: Construction of a chromosome-scale long-read reference genome assembly for potato

Version: Original Submission **Date:** 7/7/2020

Reviewer name: Jian-Feng Mao, Ph.D.

Reviewer Comments to Author:

Review: Construction of a chromosome-scale long-read reference genome assembly for potato
The authors described the updated genome assembly for potato and provided the genome annotations, especially the annotation of centromeres. The reported genome assembly represented much improvement over the released ones. This study and the associated data are very much valuable to the potato genetic and breeding communities.
While the manuscript is well written, we have a few minor comments:
1. First of all, the main text has no line numbers for reviewers. It is a little bit hard to input specific comments.
2. Would the authors like to report the ONT sequencing in depth both the main text and Tables, like Table S2?
3. For each polishing steps, would you like to report the improvement (or changes) you gained. Also, I may want to know why you chose three rounds of Pilon polishment? Why not 2 or 4 rounds?
4. Do you have any means to examine whether there are redundances of haplotigs? Do you think Redundans (https://github.com/lpryszcz/redundans) could help in this?
5. In the genome assessment section, would you like to report the heterozygosites (polymorphic sites) you called from shotgun reads alignment? This is interesting for readers.

Level of Interest

Please indicate how interesting you found the manuscript: Choose an item.

Quality of Written English

Please indicate the quality of language in the manuscript: Choose an item.

Declaration of Competing Interests

Please complete a declaration of competing interests, considering the following questions:

- Have you in the past five years received reimbursements, fees, funding, or salary from an organisation that may in any way gain or lose financially from the publication of this manuscript, either now or in the future?
- Do you hold any stocks or shares in an organisation that may in any way gain or lose financially from the publication of this manuscript, either now or in the future?

- Do you hold or are you currently applying for any patents relating to the content of the manuscript?
- Have you received reimbursements, fees, funding, or salary from an organization that holds or has applied for patents relating to the content of the manuscript?
- Do you have any other financial competing interests?
- Do you have any non-financial competing interests in relation to this paper?

If you can answer no to all of the above, write 'I declare that I have no competing interests' below. If your reply is yes to any, please give details below.

I declare that I have no competing interests

I agree to the open peer review policy of the journal. I understand that my name will be included on my report to the authors and, if the manuscript is accepted for publication, my named report including any attachments I upload will be posted on the website along with the authors' responses. I agree for my report to be made available under an Open Access Creative Commons CC-BY license (http://creativecommons.org/licenses/by/4.0/). I understand that any comments which I do not wish to be included in my named report can be included as confidential comments to the editors, which will not be published.

Choose an item.

To further support our reviewers, we have joined with Publons, where you can gain additional credit to further highlight your hard work (see: https://publons.com/journal/530/gigascience). On publication of this paper, your review will be automatically added to Publons, you can then choose whether or not to claim your Publons credit. I understand this statement.

Yes Choose an item.
