# Supplementary material for: Construction of a chromosome-scale long-read reference genome assembly for potato
Source: Gigascience. 2020 Sep 23;9(9):giaa100. doi: 10.1093/gigascience/giaa100 (PMC7509475; doi:10.1093/gigascience/giaa100)
Supplement: giaa100_Reviewer_2_Report_Original_Submission — Alexis Sullivan -- 8/10/2020 Reviewed [file giaa100_reviewer_2_report_original_submission.pdf]

Reviewer Report

Title: Construction of a chromosome-scale long-read reference genome assembly for potato

Version: Original Submission **Date:** 8/10/2020

Reviewer name: Alexis Sullivan

Reviewer Comments to Author:

Pham et al. present a reference-quality genome assembly for a doubled monoploid potato clone using Oxford Nanopore long reads and Hi-C scaffolding. Previously generated resources, including a genetic map from 190 individuals, were used to validate the placement of scaffolds onto chromosome-sized pseudomolecules. New Oxford Nanopore cDNAs and published RNA-seq libraries were used to annotate gene models, which yielded complete representation of ~93% of the BUSCO orthologs.
This new cultivated potato assembly is a considerable improvement over previous versions and will be a welcome addition to the growing number of high-quality plant genome assemblies. Overall, the manuscript is well written and organized with adequate detail to reproduce the assembly and annotations. I think the depth of analysis here is probably more than sufficient for a data note, and all figures, tables, and supplementary materials are warranted and clearly presented.
I have some minor comments on a few places where I feel additional details or clarification would be helpful:
1. Was the average size of size of the isolated high molecular weight DNA measured?
2. Please specify the pore type in the flow cell.
3. What was the average quality for the ONT reads? I am not very familiar with Guppy, but can the --qscore_filtering option be passed without also specifying --min_qscore ? If so, what is the default minimum Q Guppy uses?
4. Why was Flye chosen over other assemblers?
5. Why was the consensus VCF generated prior to Pilon polishing?
6. How many Illumina reads were used with Pilon? The ~459 million mentioned in the contiguity and accuracy section?
7. Could you elaborate on how "recombination bins [were] manually adjusted to eliminate incorrect bins"? How were these bins identified as incorrect?
8. Intact LTRs were annotated using LTRharvest, LTR_finder and LTR_retriever for assessing assembly continuity using the LAI metric. Were these identified LTRs later used in RepeatModeler to mask the assembly or included in the final custom repeat library? Which set(s) of repeats were used to soft mask the genome prior to gene prediction?
9. How much cDNA and RNA-Seq transcript data were ultimately aligned to the genome and used for gene annotation?
10. What do the green boxes in Fig S1 represent?

Level of Interest

Please indicate how interesting you found the manuscript: Choose an item.

Quality of Written English

Please indicate the quality of language in the manuscript: Choose an item.

Declaration of Competing Interests

Please complete a declaration of competing interests, considering the following questions:

- Have you in the past five years received reimbursements, fees, funding, or salary from an organisation that may in any way gain or lose financially from the publication of this manuscript, either now or in the future?
- Do you hold any stocks or shares in an organisation that may in any way gain or lose financially from the publication of this manuscript, either now or in the future?
- Do you hold or are you currently applying for any patents relating to the content of the manuscript?
- Have you received reimbursements, fees, funding, or salary from an organization that holds or has applied for patents relating to the content of the manuscript?
- Do you have any other financial competing interests?
- Do you have any non-financial competing interests in relation to this paper?

If you can answer no to all of the above, write 'I declare that I have no competing interests' below. If your reply is yes to any, please give details below.

I declare that I have no competing interests

I agree to the open peer review policy of the journal. I understand that my name will be included on my report to the authors and, if the manuscript is accepted for publication, my named report including any attachments I upload will be posted on the website along with the authors' responses. I agree for my report to be made available under an Open Access Creative Commons CC-BY license (http://creativecommons.org/licenses/by/4.0/). I understand that any comments which I do not wish to be included in my named report can be included as confidential comments to the editors, which will not be published.

Choose an item.

To further support our reviewers, we have joined with Publons, where you can gain additional credit to further highlight your hard work (see: https://publons.com/journal/530/gigascience). On publication of this paper, your review will be automatically added to Publons, you can then choose whether or not to claim your Publons credit. I understand this statement.

Yes Choose an item.
